# Prenatal Exposure to Air Pollution and Risk for Attention-Deficit/hyperactivity Disorder in Children

**DOI:** 10.1007/s10802-025-01397-9

**Published:** 2026-02-12

**Authors:** Sharanpreet Kaur, Josefa Canals-Sans, Paula Morales-Hidalgo, Mònica Guxens, Sami Petricola, Victoria Arija

**Affiliations:** 1 Nutrition and Mental Health Research Group (NUTRISAM), Universitat Rovira I Virgili (URV), Tarragona, Spain; 2 Research Center for Behavior Assessment (CRAMC), Department of Psychology, Universitat Rovira I Virgili (URV), Tarragona, Spain; 3 Institut d’Investigació Sanitària Pere Virgili (IISPV), Reus, Spain; 4 IU-RESCAT: University Research Institute On Sustainable, Climate Change and Energy Transition, Universitat Rovira I Virgili, Tarragona, Spain; 5 Instituto de Salud Global de Barcelona (ISGlobal), Barcelona, Spain; 6 Catalan Institution for Research and Advanced Studies (ICREA), Barcelona, Spain; 7 Universitat Pompeu Fabra, Barcelona, Spain; 8 Spanish Consortium for Research On Epidemiology and Public Health (CIBERESP), Instituto de Salud Carlos III, Madrid, Spain; 9 Department of Child and Adolescent Psychiatry/Psychology, Erasmus MC, University Medical Centre, Rotterdam, The Netherlands; 10 Department of Basic Medical Sciences, Universitat Rovira I Virgili, Reus, Spain

**Keywords:** Air pollution, ADHD, Pregnancy, Children, Inattention, Diagnosis

## Abstract

Exposure to air pollutants during early life may impair children’s neurodevelopment and increase the risk of ADHD. The study aimed to explore the association between exposure to prenatal air pollutants and ADHD in the school population from Tarragona, Spain. Data were obtained from the EPINED study, a two-phase epidemiological research involving preschool-aged (4–5 years) and school-age (10–11 years) children. The screening phase assessed ADHD symptoms in 3,727 participants, followed by a diagnostic phase with 781 children (549 without ADHD, 174 with ADHD). Multivariable regression showed that higher prenatal exposures to PM_10_ (β-1.88; CI: 0.65–3.12), PM_coarse_ (β-1.25; CI: 0.52–1.98), NO_2_ (β-1.11; CI: 0.56–1.66), and NO_x_ (β-1.29; CI: 0.66–1.91) were significantly associated with increased teacher-reported ADHD symptoms in school-age children. No significant association was observed between exposure to air pollutants and any ADHD presentation. Trimester-specific analyses revealed significant associations between prenatal exposure to air pollutants during early pregnancy and inattentive symptoms, as assessed by the Schedule for Affective Disorders and Schizophrenia for school-age children and teacher reports. In the first and second trimesters, exposure to PM_2.5_, PM_10_, PM_coarse_, NO_2_, and NO_x_ was significantly associated with increased inattention, highlighting critical windows of gestational susceptibility to air pollution and ADHD risk, with effects more pronounced in males. Our findings suggest that even modest increases in ADHD symptoms may reflect subtle neurodevelopmental effects of prenatal air pollution exposure. These results highlight early gestation as a vulnerable period and the need for further research on long-term impacts.

**Supplementary Information:**

The online version contains supplementary material available at 10.1007/s10802-025-01397-9.

## Introduction

Attention deficit hyperactivity disorder (ADHD) is a prevalent neurodevelopmental disorder (NDD) defined by persistent patterns of inattention and/or hyperactivity and impulsivity interfering with children’s functioning or development (APA, 2022). Globally, the prevalence of ADHD has been reported at 8% in children and adolescents, or 1.13% across the lifespan, but up to 2.5 times higher rates in males than females (Ayano et al., 2023; Cortese et al., 2023). In particular, in Spain, the ADHD prevalence among preschool-aged (4–5 years) and school-age (10–11 years) children is reported at 3.0% and 7.7% respectively, with an average of 5.5% (Canals Sans et al., 2021). A meta-analysis based on both rating scales and clinical diagnostic interviews across the lifespan reports that males exhibit significantly more severe hyperactivity/impulsivity symptoms than females (Young et al., 2024). Despite growing recognition of its public health importance, the aetiology of ADHD remains multifactorial and incompletely understood. While genetic factors play a major role, environmental exposures, particularly air pollution, have emerged as significant and modifiable determinants of neurodevelopmental risk (Kaur et al., 2023; WHO, 2024; Zeng et al., 2025; Zhao et al., 2024).

Since ADHD is a neurodevelopmental disorder, with symptom onset occurring before the age of 12 according to the DSM-5-TR (APA, 2022) and peaking at the ages between 5–9 years (Cortese et al., 2023), it is particularly important to explore the prenatal effects of air pollutants, as this period constitutes a critical window of vulnerability for the developing brain (Ha, 2021). Evidence also suggests that male foetuses are more susceptible to adverse neurodevelopmental effects of environmental exposures than females (Goodman et al., 2023; Moore, 2024). Maternal exposure to air pollutants during pregnancy may induce neuroinflammation, oxidative stress, excitotoxicity, and epigenetic modifications in the foetus (Kalenik et al., 2025). These mechanisms may contribute to long-term alterations in neural circuitry underlying attention and behavioural control. However, despite growing evidence linking air pollution to neurodevelopmental outcomes, findings regarding its association with ADHD risk remain inconsistent, with very limited studies identifying a sensitive exposure window during pregnancy, ADHD clinical presentations, and familial confounders such as parental ADHD. This represents an issue of significant clinical and public health importance, with implications for early prevention and environmental policy.

Recent systematic reviews and meta-analyses illustrate both the potential importance of this association and the limitations of the existing evidence. A meta-analysis by Abraham et al. (2025) identified only two studies suitable for inclusion on the effects of prenatal air pollution exposure on ADHD diagnosis, highlighting the need for further research to confirm these associations. Similarly, a systematic review by Kaur et al. (2023) found that while prenatal exposure to polycyclic aromatic hydrocarbons (PAH) and particulate matter (PM) was consistently associated with ADHD-related symptoms in children, data on NO_2_ and sulphur dioxide (SO_2_) remain inconsistent, and evidence on carbon monoxide (CO) and ozone (O_3_) has scarcely been investigated. In particular, it has been observed that prenatal exposure to suspended particulate matter (SPM), NO_2_ and SO_2_ was associated with behavioural problems in children, including issues with attention, aggression, and poor impulse control commonly associated with ADHD (Bae et al., 2025; Yorifuji et al., 2016, 2017). Higher exposure levels to PAH during pregnancy are associated with higher anxiety or depression, ADHD symptoms (inattention and hyperactivity) and lower cognitive development (Pagliaccio et al., 2020; Perera et al., 2012, 2014, 2018, 2024; Peterson et al., 2015). Several studies have reported that exposure to pollutants such as PM_2.5_ and PM aerodynamics of ≤ 10 μm (PM_10_), NO_2_, nitrogen oxides (NO_x_) and O_3_ is associated with lower motor function, inattention and hyperactivity symptoms in children (Iglesias-Vázquez et al., 2022; Shih et al., 2020; Zeng et al., 2025). However, other studies have found no significant association between prenatal exposure to air pollutants and the development of ADHD or related symptoms (Forns et al., 2018; Gong et al., 2014; Oudin et al., 2019). Moreover, very limited studies have explored the trimester effect on ADHD risk in children; for instance, Chang et al. (2022) found that exposure to PM_2.5_ during the first trimester was positively associated with risk of ADHD in children. While growing evidence points toward an association between prenatal air pollution and ADHD risk, inconsistencies across pollutants exposure assessment methods (fixed-site monitoring stations vs land-use regression or satellite-based models), exposure time windows (whole pregnancy vs trimester-specific analyses), outcome definitions (clinically diagnosed ADHD vs symptoms) and differing degrees of adjustment for potential confounders warrant further investigation.

Most of the existing research has been conducted in limited geographic contexts, with two of the three studies identified by Abraham et al. (2025) based in Taiwan, limiting generalisability and international applicability as well as lacking control for parental ADHD diagnoses, and none analysing ADHD clinical presentations. This gap is particularly relevant in regions characterised by intensive petrochemical activity, where pollutant exposure arises from both direct (traffic and industrial emissions of NO_x_, SO_2_, primary PM) and secondary sources (formation of O_3_, secondary PM, and other photochemical oxidants). (Rovira et al., 2020, 2021). Populations residing in these industrialised Mediterranean areas may face disproportionate exposure levels and assess their potential contribution to ADHD risk.

This study aims to investigate the association between prenatal exposure to air pollutants, including PM_2.5_, PM_10_, PM_coarse_ (PM with an aerodynamic diameter between 2.5–10 μm), PM_2.5 absorbance_ (a proxy for black carbon content in fine particulate matter), NO_2_, NO_x_, and O_3_, and the likelihood of ADHD symptoms and diagnosis in a Mediterranean area considered of high petrochemical activity. In addition, we assess ADHD clinical presentations (inattentive, hyperactive-impulsive, and combined), examine exposure by pregnancy trimester, and adjust for a wide range of covariates, including parents’ self-reported ADHD symptoms. We hypothesise that higher prenatal exposure to multiple air pollutants is significantly associated with an increased risk of ADHD symptoms in children in our region. Specifically, we expect that, first, prenatal air pollution exposure will show a stronger association with dimensional ADHD symptomatology than with categorical diagnosis, with a greater effect observed in males than females, independent of environmental and familial covariates; and, second, early gestational periods will represent a window of heightened vulnerability for the emergence of ADHD symptoms.

## Methods

### Design and Participants

**Fig. 1:**
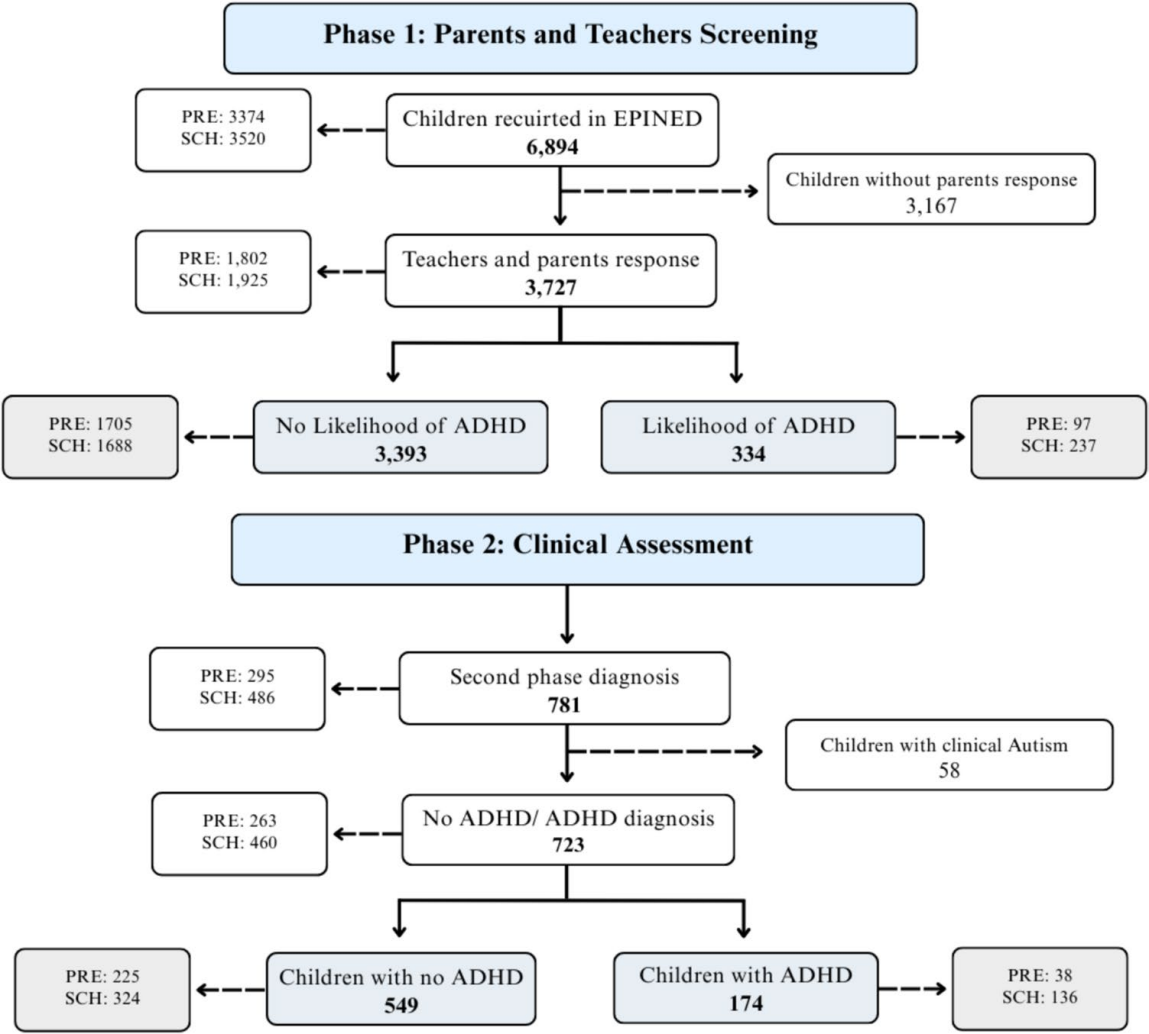
Flowchart of the children recruited in the study. Abbrev: PRE: preschool-aged children (4–5 years); SCH: school-age children (10–11 years); ADHD: attention deficit-hyperactivity disorder

The current study is part of a two-phase project, conducted between the years 2014–2019, entitled Neurodevelopmental Disorders Epidemiological Project (EPINED), in the region of Tarragona (Catalonia), a province in the north-eastern part of Spain (Fig. 1). During the first phase, a total of 6,894 children were screened for symptoms of ADHD with the aid of the CONNERS age-appropriate screening tools. Of these, 54% of families provided consent to participate, resulting in 3,727 children (1,798 males and 1,929 females; mean age = 7.29 years, SD = 3.03) from two age groups: preschool-aged (4–5 years) and school-age (10–11 years), with completed parent and teacher questionnaires, who continued in the study. Based on the screening score, 334 children exceeded the threshold, indicating a potential risk for ADHD. In the second phase, 781 children (comprising both those who screened positive and a comparison group who screened negative) were individually evaluated by two qualified psychiatrists and psychologists to confirm or rule out an ADHD diagnosis based on DSM-5 criteria. Children with a clinical diagnosis of autism were excluded from the sample (n = 58). Therefore, a total of 723 children (549 without ADHD and 174 with ADHD; 440 males and 283 females) with a mean age of 8.44 (SD 2.95) were included in the analysis. The Ethics Committee of Sant Joan University Hospital approved the study protocol (13–10–31/10proj5), and parents were informed and consented to take part. A comprehensive diagnostic procedure is described in Canals Sans et al. (2021) and Morales Hidalgo et al. (2021).

### Outcome: ADHD Symptoms and Diagnosis

ADHD symptoms were assessed by parents and teachers using age-appropriate questionnaires of Conners versions: the *Conners Early Childhood Global Index* (CONNERS EC GI) for children aged 2–6 years (preschool-aged), and the *Conners 3 ADHD Index* (CONNERS AI) for children aged 6–18 years (school-age) (Conners, 2008; Conners & Goldstein, 2009). The Likert scores were indexed as follows: 0–3, with 0 indicating “never”, 1 “occasionally”, 2 “often”, and 3 “very often”. A T-score between 65–69 represents an “elevated” level of symptoms, while a T-score ≥ 70 indicates a “highly elevated” level of symptoms. The questionnaires were adapted into Spanish, achieving internal reliability (Cronbach’s α) of 0.97 and 0.92 (Conners EC GI) or 0.98 and 0.96 (Conners AI) for teachers and parents, respectively (Morales-Hidalgo et al., 2017). Conners can be further used to assess symptoms of ADHD, such as restless-impulsive and emotional lability in preschool-aged children from Conners EC-GI, whereas hyperactive-impulsive and inattentive symptoms in school-age children from Conners AI (Kaur et al., 2024; Morales-Hidalgo et al., 2017).

Clinical information for the ADHD diagnosis was collected from the parents by the semi-structured diagnostic interview Schedule for *Affective Disorders and Schizophrenia for School-Age Children-Present and Lifetime version* (K-SADS-PL; Kaufman et al., 1997). The Spanish version has shown excellent inter-rater reliability of κ = 0.91 (Ulloa et al., 2006). A diagnosis of ADHD, inattentive, hyperactive-impulsive, or combined presentation, was established based on DSM-5 criteria, requiring the presence of at least 6 out of 9 symptoms for either the inattentive or hyperactive-impulsive presentation, along with criteria for age of onset, symptom duration, and functional impairment across multiple settings. Children were classified as having the combined presentation when diagnostic criteria for both inattention and hyperactivity-impulsivity were met concurrently. Symptom severity was determined based on K-SADS item scores; each was rated on a Likert scale from 0 to 3. Subscale scores for inattention and hyperactivity-impulsivity symptoms range from 0 to 27, and the total severity score ranges from 0 to 54, with higher scores indicating greater symptom severity. In addition to the interview, a neuropsychological assessment was conducted using the *Wechsler Preschool and Primary Scale of Intelligence* (WPPSI-IV) for preschool-aged children and the *Wechsler Intelligence Scale for Children* (WISC-IV) for school-age children (Wechsler, 2005, 2014) from which Total IQ scores were obtained.

### Estimation of Air Pollution Exposure

Parents' exposure to air pollutants during pregnancy was assessed using parent-reported residential history going back to the child's birth. A Land Use Regression (LUR) model, created for the ESCAPE (European Study of Cohorts for Air Pollution Effects) project, was used to estimate exposure to traffic-related air pollutants at birth home addresses. This model included six air pollutants: PM_2.5_ (aerodynamics of ≤ 2.5 μm), PM_10_ (aerodynamics of ≤ 10 μm), PM_coarse_ (aerodynamics between 2.5 and 10 μm), PM_2.5absorance_, an indicator of black carbon with aerodynamics of < 2.5 μm (a component of PM_2.5_), NO_2_, and NO_x_. The region's warm, cold, and intermediate temperature seasons in 2009 were covered by two weeks of measurements of PM (PM_2.5_, PM_10_) and NO_x_/NO_2_ from 20 and 40 locations, respectively (Eeftens et al., 2012). PM_2.5absorbance_ was measured in the lab as a component of PM_2.5_ particles, and PM mass between 10 and 2.5 µm (PM_coarse_) was computed by subtracting PM_2.5_ from PM_10_. In cases where monitoring data were limited, temporal exposure estimates for pollutants (PM_10_, PM_2.5_, PM_abs_, PM_coarse_, and NO_x_) were derived using NO_2_ ratios, providing a practical approach to estimate pollutant levels over time. The ELAPSE (Effects of Low-Level Air Pollution: A Study in Europe) project's O_3_ estimates were utilised (de Hoogh et al., 2018). Using the AirBase v8 dataset (EEA, 2015), daily O_3_ concentration was determined in 2010 for an 8-h maximum average per day for the warm (April–September) and cold (January–March and October–December) seasons. Based on the residential address each participant supplied throughout pregnancy, an estimated exposure to air pollution was calculated from the background monitoring and daily data obtained. Using the average annual concentration routine from the background monitoring locations, the average concentration for the whole pregnancy time was estimated using a back-extrapolation method. For the Catalonia region of Spain, the LUR model produced an R^2^ of 76% for PM_coarse_ and PM_10_, 75% for PM_2.5 abs_, 71% for NO_2_, 69.6% for O_3_, 69% for NO_x_, and 62% for PM_2.5_ (de Hoogh et al., 2018; Eeftens et al., 2012).

### Covariables

*Normalised Difference Vegetation Index* (NDVI), quantifying the vegetation greenness within the 300 m buffer zone, was calculated from the geocoded locations. (Weier & Herring, 2000). Satellite images from 2009–2015 were obtained from the USGS website (https://espa.cr.usgs.gov/) to cover the full period and were then processed to eliminate low-quality pixels, including those representing water, snow reflections, or saturation. Furthermore, the Mapa de Cobertes de Sol de Catalonia (MCSC version 4, 2009) was used to calculate the *distance to the nearest industry* using land cover classification. The *deprivation index* was measured following the Spanish census section for measuring socioeconomic deprivation (Duque et al., 2021). *Type of residential area*: urban (> 10,000), suburban (2,000–10,000) and rural (< 2,000) were identified based on the number of inhabitants living in an area, based on the Spanish Instituto Nacional de Estadística (INE) classification.

Other factors related to children and family, were also considered, such as the *age* (years) of the children at phase 1 or phase 2 of the study, *sex* (male or female) of the child, *ethnicity* if born in Spain or outside (Spanish or others), *socio-economic status* (SES: high, medium or low) of the family which was calculated based on the Catalan classification of occupations (CCO-2011). Further, covariates were selected based on their established or potential associations with neurodevelopmental outcomes and risk of confounding in studies of prenatal environmental exposures (Kian et al., 2022). We considered pregnancy-related factors of the mother, such as *toxins during pregnancy*, i.e., tobacco or alcohol consumption during pregnancy (Xavier et al., 2022), and if the child was *small for gestational age* (SGA) (Salehi et al., 2025), calculated based on the birth weight of the child and gestational age (*weeks of pregnancy*).

Additionally, parental ADHD risk was also considered to control for genetic predisposition (Uchida et al., 2023), obtained from the World Health Organisation *Adult ADHD Self-Report Scale* (ASRS; Kessler et al., 2007). The ASRS is a self-reported questionnaire administered to parents, designed to assess the current presence of ADHD symptoms according to the diagnostic criteria of the DSM-5-TR in adults. It consists of 18 items with a Likert-type response format with 5 response options, of which 9 items assess attention problems, and the other 9 items assess symptoms related to hyperactivity-impulsivity. The Spanish adaptation of the ASRS has demonstrated excellent validity and reliability, with high sensitivity (96.7%) and specificity (91.1%), strong agreement (κ = 0.88), and high accuracy (AUC = 0.94; OR = 297.3, 95% CI = 76.2–1,159) (Ramos-Quiroga et al., 2009).

### Statistical Analyses

Each air pollutant was homogenised with its respective conventional unit to allow the comparability of our results with previous studies, such as for PM_2.5_ and PM_coarse_: 5 µg/m^3^; PM_2.5abs_: 10^–5^ m^−1^, NO_2_, O_3_ and PM_10_: 10 µg/m^3^; and NO_x_: 20 µg/m^3^. To analyse the correlation between air pollutants, Spearman’s correlation was conducted. To minimise data loss, missing values were imputed using the predictive mean matching method. The data was to ensure uniformity for further analyses, the presentation scores from Conners ECGI (restless-impulsivity and emotional lability) and Conners AI (hyperactive-impulsivity and inattention) were converted to Z-scores. In contrast, the total T-scores from both Conners ECGI and Conners AI were retained in their original form to facilitate comparison with other studies. Separate multiple linear regressions were conducted to obtain β for continuous data (Conners T-scores and K-SADS severity score), and logistic regression was performed for categorical data (no diagnosis vs clinical ADHD presentations) to obtain odds ratios (ORs), both with 95% confidence levels (CIs). To control for multiple comparisons and reduce the chances of false positives, a Bonferroni correction was applied, *p* = 0.0125. This was obtained via an effective number of tests (n = 4) suggested by the principal component analysis (PCA), based on the covariates introduced in the fully adjusted model. A Directed Acyclic Graph (DAG) shows the covariates introduced in the model (Supplementary Fig. 1). Statistical analyses were performed using IBM SPSS Statistics (version 29; IBM Corp., 2022), while graphs were created in R (version 4.3.0; R Core Team, 2023) using the ggplot2 package (Wickham, 2016).

## Results

### Participants Description

The sociodemographic characteristics of participants in Phase 1 and Phase 2 are summarised in Table 1. In Phase 1, significant differences were observed between groups in terms of sex, with a higher proportion of males in the ADHD-likely group (59.9%) compared to the non-ADHD group (47.1%). Most participants were of Spanish ethnicity (84.1%). A significant difference in SES was found, with a greater proportion of participants in the ADHD-likely group classified as low SES (24.3%) compared to the non-ADHD group (15.8%). No significant differences were observed in terms of residential area, with most participants residing in urban areas (64.6%).

**Table 1:** Descriptive characteristics of participants in phases 1 and 2

Phase 1 (Screening)			No likelihood of ADHD	Likelihood of ADHD*	*p*^*ab*^
			*(N* = *3393)*	*(N* = *334)*	
Sociodemographic data					
Sex		Male	47.1 (1598)	59.9 (200)	<.001
		Female	52.9 (1795)	40.1 (134)	
Ethnicity		Spanish	84.2 (2858)	82.9 (277)	.536
		Others	15.8 (535)	17.1 (57)	
SES		Low	15.8 (535)	24.3 (81)	<.001
		Medium	58.1 (1971)	60.5 (202)	
		High	24.4 (828)	14.7 (49)	
Residential area		Urban	64.7 (2195)	65.8 (213)	.328
		Suburban	31.6 (1071)	29.2 (101)	
		Rural	3.7 (127)	4.9 (20)	
ADHD risk assessment					
CONNERS ECGI (n = 1802)			*(n* = *1705)*	*(n* = *97)*	
Age			4.21 ± 0.54		
Teachers					
Restless-impulsivity			47.41 ± 8.05	72.12 ± 7.82	<.001
Emotional Lability			47.55 ± 7.64	72.25 ± 13.15	<.001
Total T-score			47.17 ± 7.32	73.70 ± 7.79	<.001
Parents					
Restless-impulsivity			60.00 ± 13.16	78.91 ± 8.21	<.001
Emotional Lability			61.02 ± 13.93	72.23 ± 12.80	<.001
Total T-score			60.62 ± 12.41	77.87 ± 8.45	<.001
CONNERS AI¤ (n = 1925)			*(n* = *1688)*	*(n* = *237)*	
Age			10.17 ± 0.50		
Teachers					
Hyperactivity-impulsivity¤			0.85 ± 1.56	4.31 ± 3.03	<.001
Inattention¤			2.52 ± 3.61	13.70 ± 4.20	<.001
Total T-score			48.57 ± 8.99	82.19 ± 9.28	<.001
Parents					
Hyperactivity-impulsivity¤			2.47 ± 2.40	5.31 ± 2.76	<.001
Inattention¤			5.77 ± 4.76	14.58 ± 4.09	<.001
Total T-score			58.36 ± 16.39	85.54 ± 7.61	<.001
Phase 2 (Diagnosis)			Non-ADHD^c^	ADHD^d^	*p*^*cd*^
			*(N* = *549)*	*(N* = *174)*	
Sociodemographic data					
Sex		Male	58.1 (319)	69.5 (121)	.007
		Female	41.9 (230)	30.5 (53)	
Ethnicity		Spanish	82.9 (455)	86.2 (150)	.300
		Others	17.1 (94)	30.5 (53)	
SES		Low	16.9 (93)	25.3 (44)	.002
		Medium	62.7 (344)	62.6 (109)	
		High	20.4 (112)	12.1 (21)	
Residential area		Rural	6.0 (33)	6.3 (11)	.613
		Suburban	32.6 (179)	29.3 (51)	
		Urban	61.4 (337)	64.4 (112)	
Distance to industry (km)			0.38 ± 0.38	0.37 ± 0.35	.867
Maternal and children's birth details					
Small for gestational age			17.7 (97)	17.2 (30)	.917
Weeks of pregnancy			38.96 ± 2.52	38.53 ± 3.07	.063
Toxins during pregnancy		Yes	17.3 (95)	27.0 (47)	.005
		No	82.7 (454)	73.0 (127)	
ADHD assessment					
Children					
K-SADS-PL—Scores					
Inattention			12.55 ± 3.96	21.68 ± 3.74	<.001
Hyperactivity-Impulsivity			12.31 ± 4.05	19.21 ± 5.41	<.001
Total			24.86 ± 6.82	40.80 ± 5.97	<.001
Presentations					
Inattentive			-	34.5 (60)	
Hyperactive-Impulsive			-	11.5 (20)	
Combined			-	54.0 (94)	
Total IQ			101.38 ± 15.38	94.48 ± 13.16	<.001
Parents ASRS score					
Mother					
	Inattention		2.43 ± 2.46	3.36 ± 2.78	<.001
	Hyperactivity		2.95 ± 2.25	3.51 ± 2.58	.027
Father					
	Inattention		2.29 ± 2.47	2.84 ± 2.65	.074
	Hyperactivity		2.57 ± 2.31	2.74 ± 2.51	.542

Data representation: M ± SD or % (n);

*Likelihood of ADHD: screened positive for ADHD symptoms based on parents' and teachers' reports.

¤For Conners AI (School), raw scores are presented for the individual presentation. Percentile scores are only available for the total score in the Conners AI (School) assessment.

Abbrev: SES: socio-economic status; IQ: intelligence quotient; Toxics during pregnancy: alcohol and/or tobacco; CONNERS ECGI: Conners Early Childhood Global Index for preschool-aged children aged 2–6 years; CONNERS AI: Conners 3 ADHD Index for school children aged 6–18 years; K-SADS-PL: Affective Disorders and Schizophrenia for school-age children- Present and Lifetime version; ASRS: ADHD Self‐Report Scale.

Further, in Phase 2, the ADHD group had a significantly higher number of males (69.5%) compared to the non-ADHD group. More children with ADHD were exposed to alcohol and/or tobacco during pregnancy (27.0%) compared to those without ADHD. Additionally, 25.3% of children in the ADHD group were from low to medium-SES families. The ADHD group also had a significantly lower total IQ (94.48) than the non-ADHD group. Finally, mothers of children in the ADHD group had significantly higher scores for ASRS (inattention and hyperactivity) than those in the non-ADHD group. Whereas, no differences are seen for the type of residential area, and distance to industry between the two groups.

### Air Pollutants Description

Table 2 shows the descriptive statistics of air pollutants measured in the study, revealing variations in concentration levels across different pollutants. PM_2.5_, PM_10_, NO_2_, and O_3_ all exceed the World Health Organisation (WHO) recommended annual limits at certain points, indicating potential air quality concerns. Whereas for PM_2.5abs_, PM_coarse_, and NO_x_ did not have established WHO limits, they showed variability in their recorded values, particularly NO_x_, with a high standard deviation of 23.76, indicating fluctuating levels. These results suggest that particulate matter and nitrogen dioxide are of particular concern regarding air quality in the studied area. The Spearman correlation (Supplementary Fig. 2) analysis suggests a strong correlation between NO_2_ and NO_x_ (r = 0.99) and between PM_10_ and PM_2.5_ (r = 0.83), both likely due to their common sources. In contrast, a negative and moderate correlation is observed between O_3_ and other pollutants, particularly with PM_2.5_ (r = −0.48) and NO_2_ (r = −0.35), indicating that higher levels of particulate matter and nitrogen oxides are associated with lower ozone concentrations, likely due to photochemical reactions.

**Table 2:** Descriptive statistics of air pollutants

Pollutants	WHO 2021*	Min	Max	Mean	SD	Median	IQR
PM_2.5_	5	7.64	22.87	14.93	0.03	15.12	2.31
PM_10_	15	14.48	59.32	32.77	5.64	33.03	6.18
PM_2.5abs_	-	0.68	4.71	1.86	0.49	1.83	0.61
PM_coarse_	-	7.69	38.72	18.69	4.44	18.46	5.78
NO_2_	10	1.81	77.35	31.52	13.65	30.97	16.84
NO_x_	-	1.47	142.78	52.71	23.76	51.55	29.52
O_3,_	60	43.29	101.53	70.07	9.4	68.59	11.93

Units of measurement: PM_2.5_, PM_10,_ PM_coarse_, NO_2_, NO_x_, O_3_: µg/m^3^ and PM_2.5abs_: 10^–5^ m^−1^.

Abbreviations: IQR: Interquartile range; SD: standard deviation; PM_2.5_: Particulate matter with aerodynamic diameter ≤ 2.5 μm; PM_10_: Particulate matter with aerodynamic diameter ≤ 10 μm; PM_coarse_: Particulate matter 2.5–10 μm; PM_2.5abs_: Absorbance of PM < 2.5 μm; NO_2_: Nitrogen dioxide; NO_x_: Nitrogen oxides; O_3_: Ozone.

*WHO annual recommended limits.

### Prenatal Air Pollutants and ADHD Symptoms: Screening Phase

**Fig. 2:**
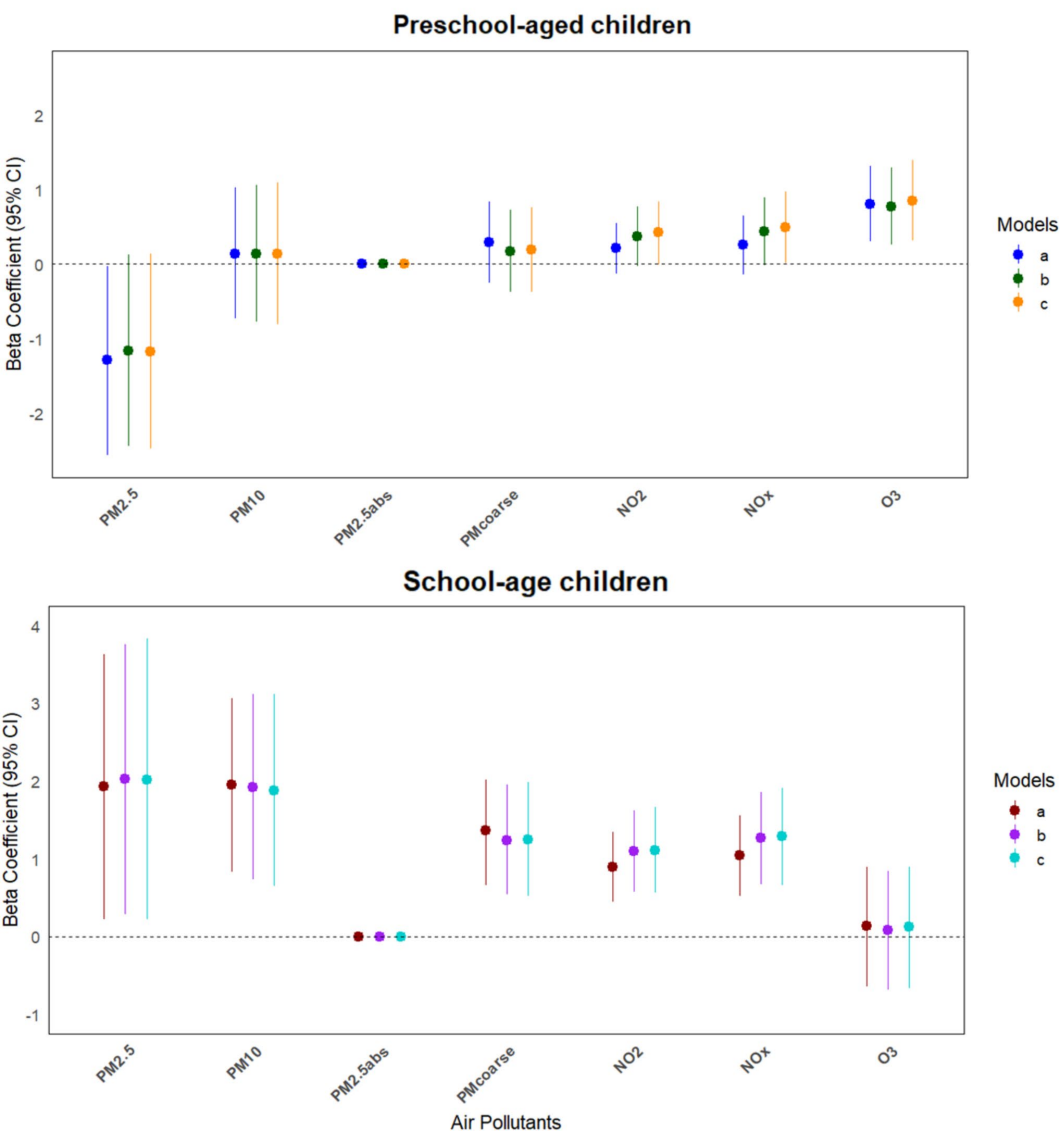
Association between prenatal air pollutants and ADHD symptoms total score reported by teachers for preschool-aged and school-age children

The multiple linear regression analysis (Fig. 2), using a *p*-value threshold of 0.012 to control for multiple comparisons, showed a significant associations across all models, both unadjusted and adjusted, between prenatal exposure to PM_10_ (β = 1.88; 95% CI: 0.65–3.12), PM_coarse_ (β = 1.25; 95% CI: 0.52–1.98), NO_2_ (β = 1.11; 95% CI: 0.56–1.66), and NO_x_ (β = 1.29; 95% CI: 0.66–1.91) and ADHD symptoms total score in school-age children as reported by teachers. There was a marginal association between PM_2.5_ and ADHD symptoms in school-age children. In contrast, a significant association was observed only for O_3_ (β = 0.85; 95% CI: 0.31–1.39) in preschool-aged children; complete βs (95% CI) can be found in Supplementary Table 1. No significant associations were observed when testing the relationship between air pollutants and ADHD symptoms total score based on parent-reported data, as detailed in Supplementary Table 2.

Furthermore, Table 3 presents multiple linear regression analyses stratified by age group and sex based on teachers' reports. For unadjusted models, PM_10_ (β = 0.17, CI: 0.04–0.29), PM_coarse_ (β = 0.10, CI: 0.02–0.19), NO_2_ (β = 0.07; CI: 0.02–0.12) and NO_x_ (β = 0.08; CI: −0.16 – 0.22) were associated with hyperactivity-impulsivity symptoms in school-age males. On the other hand, inattention in school-age males was marginally associated with PM_coarse_ and was significantly associated with NO_2_ (β = 0.11; CI: 0.05–0.17) and NO_x_ (β = 0.13; CI: 0.06–0.19), even after adjusting for confounding variables. In females, no associations were found between air pollutants and inattention. In contrast, O_3_ exposure was significantly associated with both restless-impulsivity and emotional lability symptoms in preschool-aged males (β = 0.12, CI: 0.04–0.20) and with emotional lability in females (β = 0.11, CI: 0.03–0.18). Regarding other pollutants, only PM_2.5_ showed marginal associations with emotional lability in preschool-aged males. No significant associations were observed between air pollutants and ADHD symptoms based on parents' reports, as detailed in Supplementary Table 3.

**Table 3:** Beta coefficient (β) with 95% confidence interval (CI) for the association between prenatal air pollutants and ADHD symptoms total score reported by teachers for preschool-aged and school-age children

Pollutants	Models	Preschool-aged children (N = 1802)				School-age children (N = 1925)			
		Restless-impulsivity		Emotional lability		Hyperactivity-impulsivity		Inattention	
		β* (CI 95%)*		β* (CI 95%)*		β* (CI 95%)*		β* (CI 95%)*	
		Male (n = 900)	Female (n = 902)	Male (n = 900)	Female (n = 902)	Male (n = 898)	Female (n = 1027)	Male (n = 898)	Female (n = 1027)
PM_2.5_	a	−0.11 (−0.31 – 0.08)	−0.11 (−0.28 – 0.07)	−0.22 (−0.43 – (−0.01))	−0.08 (−0.32 – 0.71)	0.14 (−0.06 – 0.35)	0.11 (−0.00 – 0.23)	0.09 (−0.09 – 0.29)	0.07 (−0.05 – 0.21)
	b	−0.13 (−0.33 – 0.07)	−0.05 (−024 – 0.13)	−0.25 (−0.46 – (−0.03))	−0.05 (−0.23 – 0.13)	0.13 (−0.08 – 0.34)	0.07 (−0.05 – 0.20)	0.11 (−0.08 – 0.31)	0.08 (−0.05 – 0.22)
	c	−0.14 (−0.35 – 0.06)	−0.05 (−0.24 – 0.14)	−0.23 (−0.45 – (−0.01))	−0.17 (−0.26 – 0.11)	0.15 (−0.07 – 0.36)	0.06 (−0.06 – 0.19)	0.17 (−0.03 – 0.37)	0.06 (−0.08 – 0.20)
PM_10_	a	−0.01 (−0.15 – 0.12)	0.09 (−0.03 – 0.22)	−0.07 (−0.21 – 0.07)	0.03 (−0.09 – 0.16)	0.17 (0.04–0.29)*	0.08 (0.01–0.16)	0.10 (−0.02 – 0.22)	0.07 (−0.01 – 0.16)
	b	−0.06 (−0.19 – 0.09)	0.13 (−0.00 – 0.26)	−0.12 (−0.27 – 0.03)	0.06 (−0.07 – 0.19)	0.15 (0.01–0.28)	0.03 (−0.05 – 0.12)	0.13 (−0.01 – 0.26)	0.06 (−0.04 – 0.15)
	c	−0.08 (−0.23 – 0.07)	0.12 (−0.01 – 0.26)	−0.11 (−0.27 – 0.05)	0.07 (−0.06 – 0.21)	0.14 (−0.01 – 0.28)	0.01 (−0.08 – 0.09)	0.15 (0.01–0.28)	0.02 (−0.07 – 0.12)
PM_2.5abs_	a	0.00 (0.00–0.00)	0.00 (0.00–0.00)	0.00 (0.00–0.00)	0.00 (0.00–0.00)	0.00 (0.00–0.00)	0.00 (0.00–0.00)	0.00 (0.00–0.00)	0.00 (0.00–0.00)
	b	0.00 (0.00–0.00)	0.00 (0.00–0.00)	0.00 (0.00–0.00)	0.00 (0.00–0.00)	0.00 (0.00–0.00)	0.00 (0.00–0.00)	0.00 (0.00–0.00)	0.00 (0.00–0.00)
	c	0.00 (0.00–0.00)	0.00 (0.00–0.00)	0.00 (0.00–0.00)	0.00 (0.00–0.00)	0.00 (0.00–0.00)	0.00 (0.00–0.00)	0.00 (0.00–0.00)	0.00 (0.00–0.00)
PM_coarse_	a	0.01 (−0.08 – 0.09)	0.08 (0.01–0.16)	−0.04 (−0.13 – 0.05)	0.04 (−0.03 – 0.12)	0.11 (0.03–0.19)*	0.04 (−0.01 – 0.08)	0.09 (0.01–0.17)	0.04 (−0.01 – 0.09)
	b	−0.02 (−0.11 – 0.07)	0.08 (0.00–0.15)	−0.07 (−0.16 – 0.03)	0.04 (−0.04 – 0.12)	0.10 (0.18–0.19)	0.02 (−0.03 – 0.07)	0.08 (0.01–0.16)	0.02 (−0.03 – 0.07)
	c	−0.02 (−0.11 – 0.07)	0.08 (0.00–0.16)	−0.06 (−0.16 – 0.04)	0.04 (−0.04 – 0.11)	0.10 (0.02–0.19)	0.02 (−0.04 – 0.07)	0.11 (0.55–0.18)	0.01 (−0.05 – 0.06)
NO_2_	a	0.03 (−0.02 – 0.08)	0.03 (−0.02 – 0.07)	−0.00 (−0.06 – 0.05)	0.02 (−0.03 – 0.06)	0.07 (0.02–0.12)*	0.05 (0.02–0.08)*	0.07 (0.02–0.12)*	0.03 (−0.01 – 0.06)
	b	0.03 (−0.03 – 0.09)	0.07 (−0.11 – 0.12)	−0.03 (−0.09 – 0.04)	0.05 (−0.01 – 0.11)	0.07 (0.01–0.13)	0.03 (−0.01 – 0.06)	0.10 (0.04–0.16)*	0.03 (−0.01 – 0.07)
	c	0.03 (−0.04 – 0.09)	0.07 (−0.01 – 0.13)	−0.02 (−0.09 – 0.05)	0.06 (−0.01—0.11)	0.06 (0.00–0.13)	0.02 (−0.02 – 0.05)	0.11 (0.05–0.17)*	0.01 (−0.03 – 0.05)
NO_x_	a	0.04 (−0.02 – 0.09)	0.03 (−0.02 – 0.09)	0.00 (−0.07 – 0.28)	0.02 (−0.04 – 0.07)	0.08 (0.16–0.22)*	0.06 (0.02–0.09)*	0.07 (0.02–0.13)	0.04 (−0.01 – 0.07)
	b	0.03 (−0.04 – 0.10)	0.08 (−0.01 – 0.14)	−0.03 (−0.11 – 0.05)	0.06 (−0.01 – 0.12)	0.07 (0.01–0.15)	0.03 (−0.01 – 0.07)	0.11 (0.04–0.18)*	0.04 (−0.01 – 0.08)
	c	0.03 (−0.04 – 0.11)	0.08 (−0.01 – 0.15)	−0.02 (−0.10 – 0.06)	0.06 (−0.00 – 0.12)	0.07 (−0.01 – 0.15)	0.02 (−0.02 – 0.06)	0.13 (0.06–0.19)*	0.02 (−0.03 – 0.07)
O_3_	a	0.08 (0.01–0.16)	0.04 (−0.04 – 0.11)	0.11 (0.03–0.19)*	0.09 (0.02–0.16)*	−0.06 (−0.15 – 0.03)	−0.01 (−0.06 – 0.05)	−0.01 (0.09–0.08)	0.03 (−0.03 – 0.09)
	b	0.09 (0.18–0.17)	0.02 (−0.06 – 0.09)	0.13 (0.05–0.21)*	0.11 (0.04–0.19)*	−0.06 (−0.15 – 0.03)	0.00 (−0.05 – 0.06)	−0.02 (−0.10 – 0.07)	0.02 (−0.04 – 0.08)
	c	0.12 (0.04–0.20)*	0.03 (−0.05 – 0.10)	0.13 (0.04–0.22)*	0.11 (0.03–0.18)*	−0.05 (−0.14 – 0.04)	0.02 (−0.04 – 0.07)	−0.01 (−0.09 – 0.08)	0.03 (−0.03 – 0.09)

Linear regression models were used to estimate β coefficients and 95% CIs.

Models (a): unadjusted; (b): adjusted for NDVI300, deprivation index, ethnicity and SES; (c): adjusted for Model b variables + distance to industry and type of residential area. Bold p = 0.05; *Multiple comparison p threshold = 0.012.

The standardized increments for each exposure are as follows: 20 µg/m^3^ for NOx, 10 µg/m^3^ for NO_2_, PM_10_, and O_3_, 5 µg/m^3^ for PM_coarse_ and PM_2.5_, and 10⁻^5^ m⁻^1^ for PM_2.5 abs_.

Abbreviations: PM_2.5_: Particulate matter with aerodynamic diameter ≤ 2.5 μm; PM_10_: Particulate matter with aerodynamic diameter ≤ 10 μm; PM_coarse_: Particulate matter 2.5–10 μm; PM_2.5abs_: Absorbance of PM < 2.5 μm; NO_2_: Nitrogen dioxide; NO_x_: Nitrogen oxides; O_3_: Ozone.

### Prenatal Air Pollutants Association and ADHD: Diagnostic Phase

**Fig. 3:**
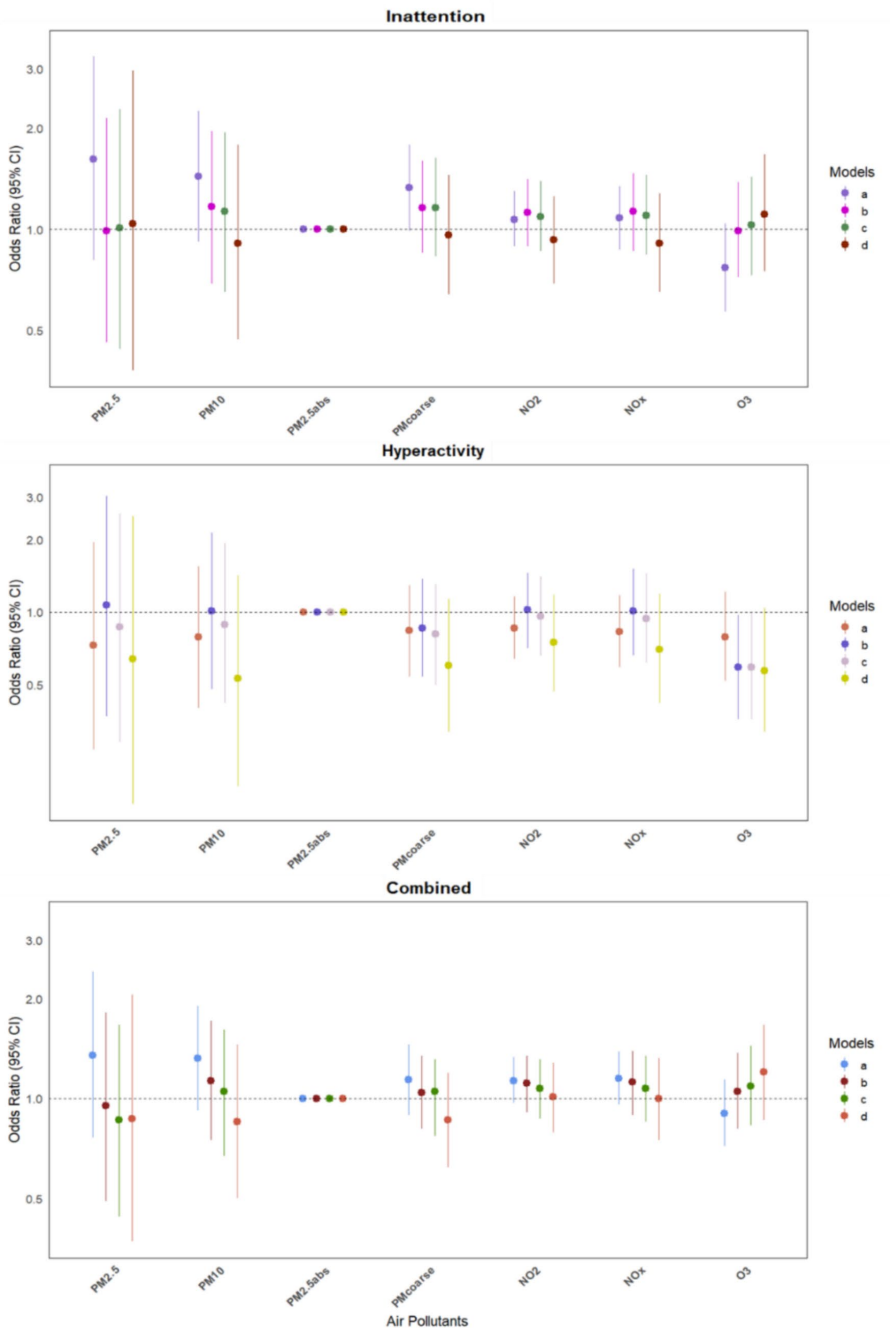
Association between prenatal exposure to air pollutants and ADHD presentations. Each pollutant was analysed in an independent regression model. Models (a): unadjusted; (b): adjusted for NDVI300, deprivation index, age, sex, ethnicity and SES; (c): adjusted for Model b variables + distance to industry and type of residential area, toxins during pregnancy, low birth weight, weeks of pregnancy; (d): adjusted for Model c variables + ASRS risk. The standardised increments for each exposure are as follows: 20 µg/m^3^ for NOx, 10 µg/m^3^ for NO_2_, PM_10_, and O_3_, 5 µg/m^3^ for PM_coarse_ and PM_2.5_, and 10⁻^5^ m⁻^1^ for PM_2.5 abs._ Abbreviations: PM_2.5_: Particulate matter with aerodynamic diameter ≤ 2.5 μm; PM_10_: Particulate matter with aerodynamic diameter ≤ 10 μm; PM_coarse_: Particulate matter 2.5–10 μm; PM_2.5abs_: Absorbance of PM < 2.5 μm; NO_2_: Nitrogen dioxide; NO_x_: Nitrogen oxides; O_3_: Ozone

Figure 3 illustrates the ORs with 95% CIs for the association between prenatal air pollution exposure and ADHD presentations. No significant associations were found between air pollutants and any of the ADHD presentations; details can be found in Supplementary Table 4. In the fully adjusted analyses, age emerged as a significant predictor only for the inattentive presentation, suggesting that inattentive symptoms may become more prominent with increasing age. Whereas it did not affect the hyperactive or combined presentations, and sex was not a significant covariate across any of the ADHD models.

### Effects of Prenatal Air Pollutants on ADHD Inattention and Hyperactivity Symptoms by Trimesters

Table 4 presents β (95% CI) for the associations between prenatal exposure to air pollutants and ADHD inattention scores reported by teachers (Conners AI) across pregnancy trimesters and the entire pregnancy period. In fully adjusted Conners models, first-trimester exposures to PM_2.5_ (β = 0.15; 95% CI: 0.07–0.23), PM_10_ (β = 0.11; 95% CI: 0.04–0.17), PM_coarse_ (β = 0.07; 95% CI: 0.03–0.11), NO_2_ (β = 0.06; 95% CI: 0.03–0.09) and NO_x_ (β = 0.07; 95% CI: 0.03–0.11) were significantly associated with higher inattention scores for school-age children and in the second trimester both NO_2_ (β = 0.04; 95% CI: 0.01–0.08) and NO_x_ (β = 0.05; 95% CI: 0.01–0.08) also remained significant. No significant associations were found for hyperactivity-impulsivity. On the other hand, no significant associations were observed between prenatal exposure to air pollutants and ADHD, restless-impulsivity and emotional lability scores in pre-school-aged children from the CONNERS ECGI. Furthermore, based on the K-SADS diagnostic interview, the inattention severity score is present in Table 5. In trimester 1, only in crude model for PM_2.5_, a significant association was observed (β = 1.12; CI: 0.38–1.86), whereas, after adjusting for confounding variables, significant associations were found for PM_10_ (β = 0.77; CI: 0.18–1.36), PM_coarse_ (β = 0.51; CI: 0.11–0.91), NO_2_ (β = 0.49; CI: 0.17–0.81), and NO_x_ (β = 0.53; CI: 0.16–0.90). In the case of O_3_ (β = 0.32: CI: −0.53 – (−0.11)), a significant and negative association is observed. In trimester 2, significant associations were observed for NO_2_ (β = 0.44; CI: 0.12–0.76) and NO_x_ (β = 0.48; CI: 0.12–0.85). However, no significant associations were observed for inattention in the third trimester. Similarly, in fully adjusted K-SADS models of hyperactivity-impulsivity, no pollutant exposure remained significant after Bonferroni correction in any trimester or across the entire pregnancy (Supplementary tables 5 and 6). Sex was observed as a significant covariate in the inattention K-SADS and CONNERS models; the association was significant only among males.

**Table 4:** Beta coefficient (β) with 95% confidence interval (CI) for the association between prenatal air pollutants and ADHD inattention score per trimester from Conners (TEACHERS)

Pollutants	*Models*	Trimester 1	Trimester 2	Trimester 3	Entire Pregnancy
		*β (CI 95%)*	*β (CI 95%)*	*β (CI 95%)*	*β (CI 95%)*
PM_2.5_	a	0.09 (0.01–0.17)	0.04 (−0.04 – 0.12)	−0.03 (−0.11 – 0.05)	0.08 (−0.04 – 0.19)
	b	0.14 (0.06–0.22)*	0.04 (−0.04 – 0.11)	−0.04 (−0.12 – 0.03)	0.09 (−0.02 – 0.21)
	c	0.15 (0.07–0.23)*	0.04 (−0.03 – 0.12)	−0.04 (−0.12 – 0.03)	0.11 (−0.01 – 0.23)
PM_10_	a	0.09 (0.03–0.15)*	0.06 (0.01–0.12)	0.01 (−0.05 – 0.07)	0.09 (0.02—0.17)
	b	0.11 (0.05–0.17)*	0.04 (−0.02 – 0.10)	−0.02 (−0.08 – 0.04)	0.08 (−0.01 – 0.16)
	c	0.11 (0.04–0.17)*	0.04 (−0.03 – 0.10)	−0.02 (−0.08 – 0.04)	0.07 (−0.01 – 0.16)
PM_2.5abs_	a	0.00 (0.00–0.00)	0.00 (0.00–0.00)	0.00 (0.00–0.00)	0.00 (0.00–0.00)
	b	0.00 (0.00–0.00)	0.00 (0.00–0.00)	0.00 (0.00–0.00)	0.00 (0.00–0.00)
	c	0.00 (0.00–0.00)	0.00 (0.00–0.00)	0.00 (0.00–0.00)	0.00 (0.00–0.00)
PM_coarse_	a	0.06 (0.02–0.11)*	0.05 (0.01–0.08)	0.02 (−0.02 – 0.06)	0.06 (0.01–0.11)
	b	0.07 (0.02–0.11)*	0.03 (−0.01 – 0.07)	0.00 (−0.04 – 0.04)	0.05 (−0.01 – 0.09)
	c	0.07 (0.03–0.11)*	0.03 (−0.01 – 0.07)	0.01 (−0.04 – 0.04)	0.05 (0.00–0.10)
NO_2_	a	0.04 (0.01–0.07)*	0.04 (0.01–0.07)*	0.03 (−0.01 – 0.06)	0.04 (0.01–0.07)*
	b	0.06 (0.03–0.09)*	0.05 (0.01–0.08)*	0.03 (−0.01 – 0.07)	0.05 (0.02–0.09)*
	c	0.06 (0.03–0.09)*	0.04 (0.01–0.08)*	0.03 (−0.01 – 0.06)	0.05 (0.02–0.09)*
NO_x_	a	0.05 (0.02–0.08)*	0.05 (0.01–0.08)*	0.04 (0.00–0.07)	0.05 (0.01–0.08)*
	b	0.07 (0.03–0.11)*	0.05 (0.01–0.09)*	0.04 (0.01–0.07)	0.06 (0.02–0.10)*
	c	0.07 (0.03–0.11)*	0.05 (0.01–0.09)*	0.04 (−0.01 – 0.07)	0.06 (0.02–0.10)*
O_3_	a	−0.02 (−0.05 – (−0.01))	0.01 (−0.01 – 0.03)	0.02 (−0.01 – 0.04)	0.01 (−0.05 −0.06)
	b	−0.03 (−0.05—(−0.01))*	0.02 (−0.01 – 0.04)	0.03 (0.01–0.05)	0.01 (−0.04 – 0.06)
	c	−0.03 (−0.05 – (−0.01))*	0.02 (−0.01 – 0.04)	0.03 (0.01–0.05)	0.02 (−0.03 – 0.07)

Each pollutant was analysed in an independent regression model.

Models (a): unadjusted; (b): adjusted for NDVI300, deprivation index, ethnicity and SES; (c): adjusted for Model b variables + distance to industry and type of residential area. Bold p= 0.05; *Multiple comparison p threshold= 0.012

The standardised increments for each exposure are as follows: 20 µg/m³ for NOx, 10 µg/m³ for NO_2_,PM_10_,and O_3_,5µg/m³ for PM_coarse_and PM_2.5_, and 10⁻⁵ m⁻¹ for PM_2.5 abs._

Abbreviations: PM_2.5_: Particulate matter with aerodynamic diameter ≤ 2.5 μm; PM_10_: Particulate matter with aerodynamic diameter ≤ 10 μm; PM_coarse_: Particulate matter 2.5–10 μm; PM_2.5abs_: Absorbance of PM < 2.5 μm; NO_2_: Nitrogen dioxide; NO_x_: Nitrogen oxides; O_3_: Ozone.

**Table 5:** Beta coefficient (β) with 95% confidence interval (CI) for the association between prenatal air pollutants and ADHD inattentive severity score per trimester from K-SADS

Pollutants	*Models*	Trimester 1	Trimester 2	Trimester 3	Entire Pregnancy
		*β (CI 95%)*	*β (CI 95%)*	*β (CI 95%)*	*β (CI 95%)*
PM_2.5_	a	1.12 (0.38–1.86)*	0.79 (0.07–1.50)	0.26 (−0.44 – 0.96)	1.33 (0.32–2.35)*
	b	0.59 (−0.16 – 1.34)	0.23 (−0.48 – 0.95)	−0.27 (−0.97 – 0.42)	0.21 (−0.86 – 1.23)
	c	0.64 (−0.13 – 1.41)	0.25 (−0.47 – 0.97)	−0.31 (−1.02 – 0.38)	0.23 (−0.88 – 1.34)
	d	0.43 (−0.56 – 1.43)	−0.02 (−0.96 – 0.91)	−0.05 (−1.43 – 0.34)	−0.33 (−1.79 – 1.13)
PM_10_	a	1.03 (0.48–1.57)*	0.80 (0.26–1.34)*	0.45 (−0.09 – 1.00)	1.14 (0.47–1.81)*
	b	0.76 (0.19–1.34)*	0.47 (−0.09 – 1.04)	0.09 (−0.45 – 0.65)	0.63 (−0.10 – 1.35)
	c	0.77 (0.18–1.36)*	0.45 (−0.12 – 1.02)	0.04 (−0.53 – 0.61)	0.60 (−0.16 – 1.36)
	d	0.40 (−0.35 – 1.16)	0.07 (−0.66 – 0.82)	−0.36 (−1.07 – 0.36)	0.01 (−0.97 – 0.99)
PM_2.5abs_	a	0.00 (0.00–0.00)	0.00 (0.00–0.00)	0.00 (0.00–0.00)	0.00 (0.00–0.00)
	b	0.00 (0.00–0.00)	0.00 (0.00–0.00)	0.00 (0.00–0.00)	0.00 (0.00–0.00)
	c	0.00 (0.00–0.00)	0.00 (0.00–0.00)	0.00 (0.00–0.00)	0.00 (0.00–0.00)
	d	0.00 (0.00–0.00)	0.00 (0.00–0.00)	0.00 (0.00–0.00)	0.00 (0.00–0.00)
PM_coarse_	a	0.70 (0.32–1.09)*	0.59 (0.21–0.97)*	0.40 (0.01–0.79)	0.71 (0.27–1.14)*
	b	0.49 (0.10–0.88)*	0.35 (−0.03 – 0.74)	0.15 (−0.23 – 0.54)	0.37 (−0.08 – 0.81)
	c	0.51 (0.11–0.91)*	0.36 (−0.03 – 0.75)	0.14 (−0.25 – 0.53)	0.37 (−0.09 – 1.27)
	d	0.25 (−0.27 – 0.77)	0.08 (−0.43 – 0.59)	−0.19 (−0.70 – 0.32)	0.03 (−0.56 – 0.64)
NO_2_	a	0.43 (0.15–0.70)*	0.42 (0.15–0.69)*	0.33 (0.05–0.62)	0.42 (0.13–0.71)*
	b	0.51 (0.19–0.81)*	0.46 (0.15–0.77)*	0.35 (0.03–0.66)	0.45 (0.12–0.78)*
	c	0.49 (0.17–0.81)*	0.44 (0.12–0.76)*	0.31 (−0.01 – 0.64)	0.42 (0.07–0.77)
	d	0.41 (−0.01 – 0.82)	0.33 (−0.07 – 0.75)	0.17 (−0.23 – 0.58)	0.34 (−0.09 – 0.78)
NO_x_	a	0.47 (0.16–0.79)*	0.47 (0.15–0.79)*	0.37 (0.04–0.70)	0.47 (0.13–0.80)*
	b	0.55 (0.19–0.91)*	0.51 (0.16–0.87)*	0.39 (0.35–0.76)	0.49 (0.11–0.87)*
	c	0.53 (0.16–0.90)*	0.48 (0.12–0.85)*	0.35 (−0.03 – 0.73)	0.45 (0.05–0.85)
	d	0.47 (−0.03 – 0.92)	0.36 (−0.11 – 0.84)	0.19 (−0.28 – 0.66)	0.36 (−0.14 – 0.88)
O_3_	a	−0.44 (−0.66 – (−0.23))*	−0.14 (−0.37 – 0.89)	−0.01 (−0.23 – 0.21)	−0.74 (−1.16 – (−0.31))*
	b	−0.33 (−0.55 – (−0.12))*	0.03 (−0.19 – 0.26)	0.13 (−0.08 – 0.34)	−0.17 (−0.63 – 0.29)
	c	−0.32 (−0.53 – (−0.11))*	0.04 (−0.19 – 0.26)	0.15 (−0.07 – 0.36)	−0.10 (−0.57 – 0.37)
	d	−0.29 (−0.55 – 0.02)	0.06 (−0.23 – 0.35)	0.16 (−0.11 – 0.43)	−0.08 (−0.67 – 0.51)

Each pollutant was analysed in an independent regression model.

Models (a): unadjusted; (b): adjusted for NDVI300, deprivation index, age, sex, ethnicity and SES; (c): adjusted for Model b variables + distance to industry and type of residential area, toxins during pregnancy, low birth weight, weeks of pregnancy; (d): adjusted for Model c variables + ASRS risk. Bold p= 0.05; *Multiple comparison p threshold= 0.012.

The standardised increments for each exposure are as follows: 20 µg/m³ for NOx, 10 µg/m³ for NO2, PM10, and O3, 5 µg/m³ for PMcoarse and PM2.5, and 10⁻⁵ m⁻¹ for PM2.5 abs.

Abbreviations: PM2.5: Particulate matter with aerodynamic diameter ≤ 2.5 μm; PM10: Particulate matter with aerodynamic diameter ≤ 10 μm; PMcoarse: Particulate matter 2.5–10 μm; PM2.5abs: Absorbance of PM < 2.5 μm; NO2: Nitrogen dioxide; NOx: Nitrogen oxides; O3: Ozone

## Discussion

In this study, we examined the association between prenatal exposure to air pollutants and ADHD in a region heavily influenced by the petrochemical industry, considering both the symptoms as reported by teachers and parents and the clinical presentations of the diagnosis. Our findings suggest the effect of prenatal air pollution exposure can be more pronounced in school-age children with ADHD than preschool-aged children, particularly to PM (PM_2.5_, PM_10_, and PM_coarse_) and nitrogen oxides (NO_2_, NO_x_) and O_3_ contributes to subtle but measurable greater in ADHD symptomatology, with effects depending on age, sex, and timing of exposure. Although the observed increases in symptom scores were modest and not consistently clinically apparent, they indicate that prenatal exposure to air pollutants may play a role in neurodevelopmental changes that affect children’s behaviour and development.

During the screening phase, prenatal exposure to PM_10_, PM_coarse_, NO_2_, and NO_x_ was significantly associated with higher ADHD total scores in school-age children based on teacher reports. For instance, each 10 or 20 µg/m^3^ increase in NO_2_ or NO_x_, respectively, during pregnancy, corresponded to approximately a rise of 0.11–0.13 Conners’ inattention scores in males in school-age children. Whereas, in preschool-aged children, O_3_ exposure showed distinct effects: each 10 µg/m^3^ increase was associated with approximately 0.11–0.12-point higher emotional lability (in females) and restless-impulsivity scores (in males), respectively. Furthermore, when stratified by trimesters, each 5–10 µg/m^3^ increase in PM_2.5_, PM_10_, and PM_coarse_ during the first trimester was associated with greater 0.15, 0.11, and 0.07 points in inattention scores, respectively.

During the diagnostic phase, no significant associations were found between pollutant exposure and categorical presentations of ADHD. However, trimester-specific analyses of inattention severity (K-SADS) revealed that first and second trimester exposures to PM_10_, PM_coarse_, NO_2_, and NO_x_ were associated with up to 0.5–0.8-point greater in inattention severity scores after adjustment, suggesting early gestation may represent a critical window for pollutant-related neurodevelopmental effects. Notably, associations were more evident in teacher reports than in parent reports, aligning with prior findings showing teachers’ higher sensitivity and specificity in identifying attention-related difficulties (Kaur et al., 2024; Morales-Hidalgo et al., 2017).

### Age and Sex Differences in Symptomatology

The observed age-related differences may reflect developmental changes in how neurodevelopmental outcomes manifest and are reported. During early childhood, rapid brain development, particularly in regions governing emotion and sensory processing, coincides with increased susceptibility to environmental stressors (Dunn et al., 2019; Lawal et al., 2022; Tierney & Nelson, 2009). Exposure to pollutants such as O_3_ has been associated with ADHD-related symptoms in children (Zhou et al., 2023). In our study, in contrast to Liu et al., (2022a, 2022b), prenatal O_3_ has been associated mainly with emotional lability in preschool-aged children, which can be a transdiagnostic manifestation, not only specific to ADHD. However, the effect was also observed on hyperactivity-impulsivity, but only in males. Yet only, few studies have explored the association between O_3_ exposure and ADHD or other NDDs. This observed pattern may reflect pollutant-related alterations in emotion-regulatory networks that are undergoing rapid maturation during this developmental window. Although the brain remains sensitive to oxidative stress and neuroinflammation throughout childhood, these effects may manifest differently as developmental trajectories of neural systems diverge and behavioural demands evolve.

In contrast, school-age children face greater cognitive and behavioural demands, and as executive functions mature, such as working memory, attentional control, and ADHD-related symptoms become more recognisable (Cortese et al., 2023). This aligns with our findings of a significant association between prenatal exposure to PM (PM_2.5_, PM_10_, PM_coarse_) and nitrogen oxides (NO_2_, NO_x_). At this stage, inflammatory processes and oxidative stress may interfere with the development of the prefrontal cortex, critical for sustained attention, impulse control, and problem-solving, making deficits in executive function, driven by prenatal air pollution exposure, more apparent as cognitive demands increase (Nimmapirat et al., 2024). Although limited studies are exploring the difference according to the age group, our results are consistent with previous research associating prenatal exposure to nitrogen oxides and PM with ADHD symptoms in children (Abraham et al., 2025; Fuertes et al., 2016; Shih et al., 2020; Yorifuji et al., 2016). Therefore, our findings could likely reflect developmental shifts in the expression and detectability of pollutant-related effects, rather than differences in underlying neurotoxicity or susceptibility.

Regarding sex differences, we have observed that prenatal air pollutants were significantly associated with inattention symptoms predominantly in school-age males. This may relate to known biological differences in vulnerability to environmental stressors (Bölte et al., 2023; Martin, 2024) and evidence that males exhibit higher sensitivity to PM_2.5_ exposure during the second trimester (Chen et al., 2020). Exposure to air pollutants during the pregnancy stage has been associated with higher anxiety levels, cognitive functioning impairments, and lower inflammatory and growth factor levels, which may contribute to behaviour change, hence compromising the neural integrity of young males (Nephew et al., 2020).

### Critical Window of Vulnerability

Our findings indicate that early gestation, particularly the first and second trimesters, may be critical periods during which prenatal exposure to ambient pollutants (PM_2.5_, PM_10_, PM_coarse_, NO_2_, and NO_x_) could influence neurodevelopmental pathways related to attention regulation. These results resonate with evidence from Chang et al. (2022), showing that first-trimester exposure to PM_2.5_ was associated with ADHD behaviours in 5-year-olds. However, some studies did not find any significant association between PM_2.5_ and any behavioural outcome related to ADHD severity and hyperactivity symptoms (Mortamais et al., 2019; Peterson et al., 2015). The consistent associations with nitrogen-based pollutants (NO_2_ and NO_x_) during the first two trimesters further underscore the potential role of traffic-related air pollution in disrupting early neural pathways involved in attentional regulation.

During these early stages, fundamental neurodevelopmental processes, such as neuronal proliferation, migration, and the second trimester, particularly marked by synaptogenesis, occur rapidly, rendering the foetal brain highly sensitive to environmental exposures (Cortes-Albornoz et al., 2023; Morgan et al., 2023). Airborne particles and gases (PMs, Nos, and PAHs) can cross the placental barrier and enter foetal circulation, potentially disrupting nutrient exchange, oxygenation, and neural development. These exposures may induce biological responses involving oxidative stress, neuroinflammation, and epigenetic alterations, which together could interfere with normal neurodevelopmental trajectories (Ha, 2021; Kalenik et al., 2025; Thangavel et al., 2022). This cascade of possible effects can compromise nutrient and oxygen exchange in the foetus, leading to foetal hypoxia and an increased risk of cognitive deficits and behavioural disorders (Liu et al., 2022a, 2022b). This has also been evidenced by imaging studies, exposure to PM_2.5_ may modify foetal brain structure, targeting the corpus callosum and lateral ventricles, and diminish cortical blood flow, affecting regions that regulate thought, emotion, and behaviour in school-age children (Mortamais et al., 2019; Peterson et al., 2015).

### Strengths and limitations

Our study had several key strengths. First, every child was screened based on teacher and parents' reports and underwent direct, individualised, clinician-administered diagnostic evaluation, avoiding the misclassification risks inherent in registry-based or electronic health records. Second, we leveraged high-resolution spatiotemporal models to estimate maternal prenatal exposure to a suite of air pollutants (NOs, PMs, O_3_), linking these estimates to each mother’s residential address and adjusting for ADHD-relevant covariates such as maternal health and pregnancy characteristics (e.g., smoking status, gestational age, SGA) as well as parental ADHD risk. Finally, by examining pollutant exposures both averaged over the full gestational period and within each trimester, our analysis captures critical windows of neurodevelopmental vulnerability and the potential synergistic effects of pollutant mixtures.

Despite these strengths, our study has several limitations. First, although we assessed multiple air pollutants, we could not include PAHs, which also play a significant role in children’s neurodevelopment. Second, our exposure estimates were based solely on maternal residential addresses and did not account for time–activity patterns, such as commuting or workplace exposures, which could potentially lead to exposure misclassification. Additionally, information on maternal occupational exposures, stress, diet, and other perinatal complications beyond SGA, tobacco, and alcohol was not available, which may have introduced residual confounding. Third, due to limited monitoring data, temporal extrapolation for several pollutants (PM_10_, PM_2.5_, PM_abs_, PM_coarse_, and NO_x_) relied on NO_2_ ratios, which may not fully capture pollutant-specific variability and could introduce additional exposure misclassification. Additionally, although we observed a significant association between air pollutant exposure and ADHD symptoms, it is important to note that the magnitude of these associations, as reflected in the ADHD symptom scale scores, may be of limited clinical relevance. Nevertheless, this finding suggests that the effects of pollutants might not be immediately apparent but could still exert subtle influences on child neurodevelopment over time.

Therefore, future studies should consider incorporating a wider range of pollutants, including PAHs and those with limited monitoring data, alongside larger sample sizes and finer spatial resolution to improve exposure assessment.

## Conclusion

Our screening phase demonstrated that prenatal exposure to PM_2.5,_ PM_10_, PM_coarse,_ NO_2_, and NO_x_ may be associated with elevated overall ADHD symptom scores in school-age children, as observed based on the teachers' reports, particularly during the first and second trimesters of pregnancy. Although no association was found for any ADHD diagnostic presentation, prenatal exposure to PM_coarse_, NO_2_ and NO_x_ during early to mid-pregnancy appeared to be associated with severity of inattention symptoms, particularly in males. While the observed higher levels in symptom scores may be modest and not always clinically apparent, they suggest that prenatal pollutant exposure could contribute to neurodevelopmental changes that influence child development over time. Together, these findings highlight potential critical windows of gestational susceptibility to air pollution and the risk of developing ADHD, with possible sex-specific effects, and underscore the need for future research to clarify the long-term impact of early-life pollutant exposure on children’s neurodevelopment.

## Conflict of interest

The authors declare that they have no conflict of interest.

## Supplementary Information

Below is the link to the electronic supplementary material.

Supplementary file1 (DOCX 190 KB)

## Data Availability

Due to legal reasons and the ethical permit for the study, the data that support the findings of this study are not publicly available. The data are accessible on reasonable request from the corresponding authors.
