# Supplementary material for: Prenatal Exposure to Air Pollution and Risk for Attention-Deficit/hyperactivity Disorder in Children
Source: Res Child Adolesc Psychopathol. 2026 Feb 12;54(1):29. doi: 10.1007/s10802-025-01397-9 (PMC12894195; doi:10.1007/s10802-025-01397-9)
Supplement: Supplementary file 1 — Supplementary file1 (DOCX 190 KB) [file 10802_2025_1397_MOESM1_ESM.docx]

**SUPPLEMENTARY INFORMATION**

**PRENATAL EXPOSURE TO AIR POLLUTION AND RISK FOR ATTENTION-DEFICIT/HYPERACTIVITY DISORDER IN CHILDREN**

| **Figure 1**: Directed Acyclic Graph (DAG) of Covariates for Air Pollutants and ADHD in children |
| --- |
| 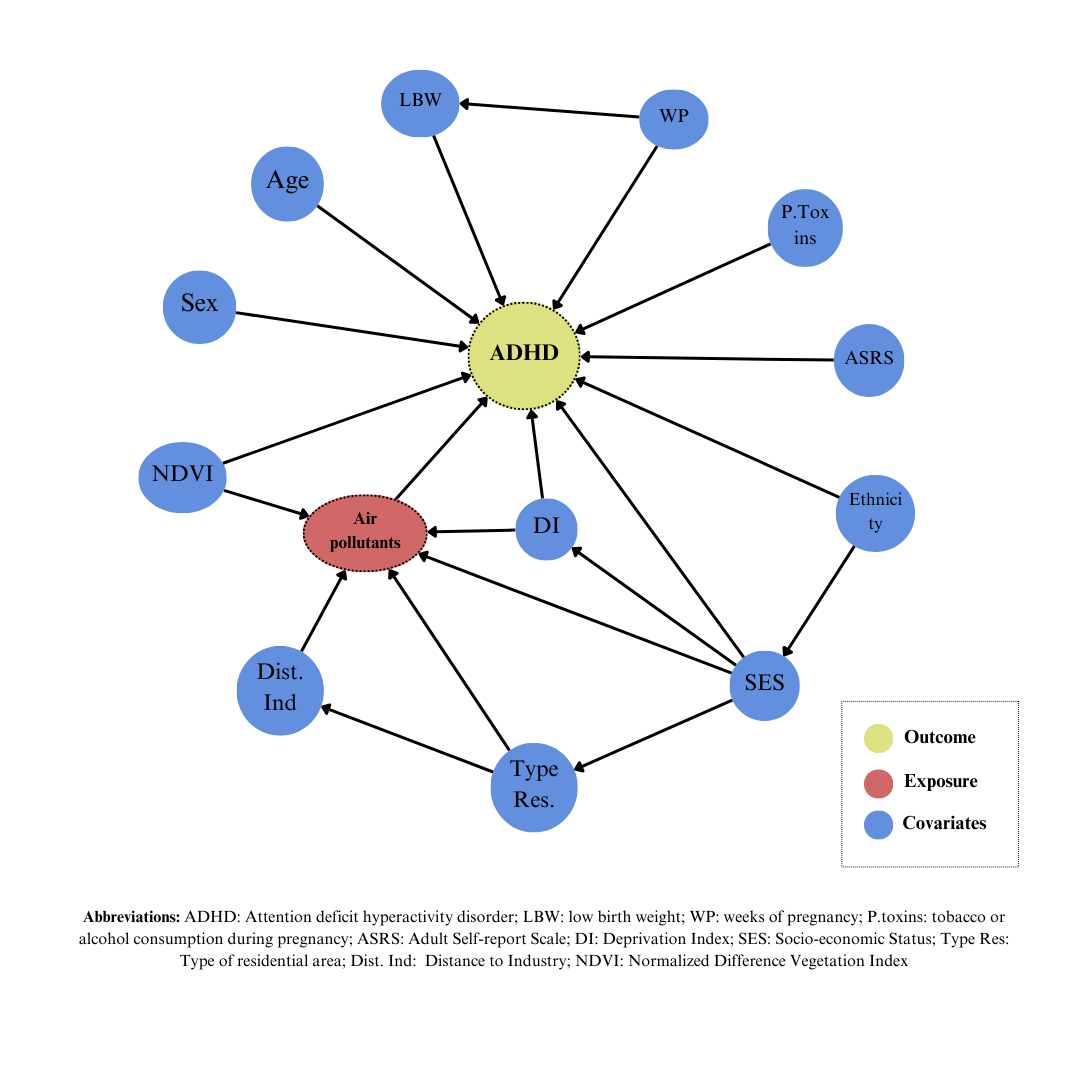 |
| **Abbreviations:** ADHD: Attention deficit hyperactivity disorder; LBW: low birth weight; WP: weeks of pregnancy; P.toxins: tobacco or alcohol consumption during pregnancy; ASRS: Adult Self-report Scale; DI: Deprivation Index; SES: Socio-economic Status; Type Res: Type of residential area; Dist. Ind: Distance to Industry; NDVI: Normalized Difference Vegetation Index |

| **Figure 2:** Spearman correlation between air pollutants |
| --- |
| **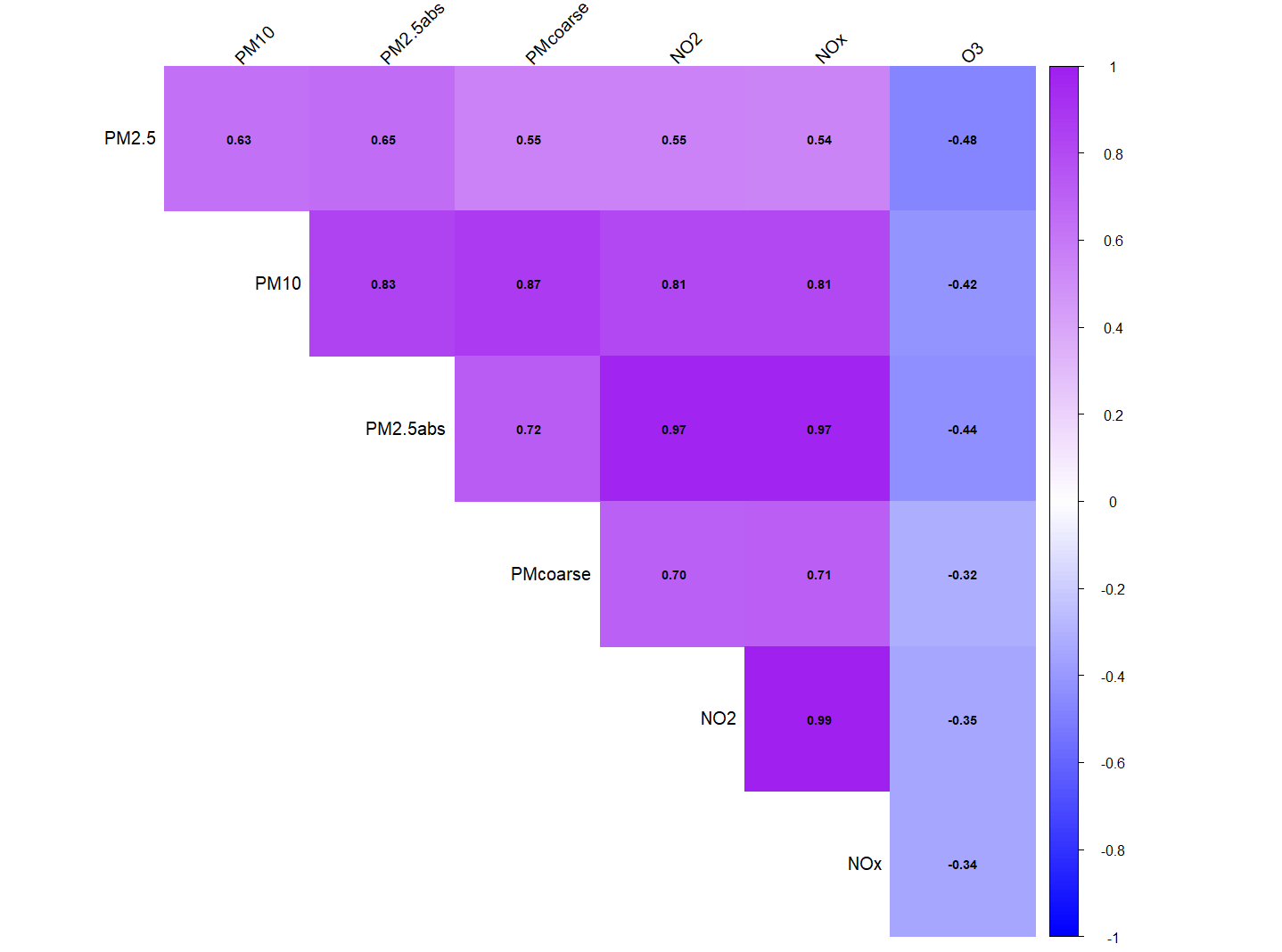** |
| **Abbreviations**: PM_2.5_: Particulate matter with aerodynamic diameter ≤ 2.5 μm; PM_10_: Particulate matter with aerodynamic diameter ≤ 10 μm; PM_coarse_: Particulate matter 2.5–10 μm; PM_2.5abs_: Absorbance of PM < 2.5 μm; NO_2_: Nitrogen dioxide; NO_x_: Nitrogen oxides; O_3_: Ozone. |

| **Table 1:** Beta coefficient (β) with 95% confidence interval (CI) for the association between prenatal air pollutants and ADHD symptoms total score reported by *teachers* for preschool-aged and school-age children | | | |
| --- | --- | --- | --- |
| **Pollutants** | **Models** | **Preschool-aged children** | **School-age children** |
|  |  | **β *(CI 95%)*** | **β *(CI 95%)*** |
| **PM_2.5_** | a | **-1.29 (-2.56 – (-0.03))** | **1.93 (0.22 – 3.63)** |
|  | b | -1.16 (-2.44 – 0.13) | **2.03 (0.29 – 3.76)** |
|  | c | -1.17 (-2.48 – 0.14) | **2.02 (0.22 – 3.83)** |
| **PM_10_** | a | 0.14 (-0.73 – 1.02) | **1.95 (0.83 – 3.06)*** |
|  | b | 0.14 (-0.78 – 1.06) | **1.92 (0.73 – 3.11)*** |
|  | c | 0.14 (-0.81 – 1.09) | **1.88 (0.65 – 3.12)*** |
| **PM_2.5abs_** | a | 0.00 (0.00 – 0.00) | 0.00 (0.00 – 0.00) |
|  | b | 0.00 (0.00 – 0.00) | 0.00 (0.00 – 0.00) |
|  | c | 0.00 (0.00 – 0.00) | 0.00 (0.00 – 0.00) |
| **PM_coarse_** | a | 0.29 (-0.25 – 0.84) | **1.36 (0.66 – 2.01)*** |
|  | b | 0.17 (-0.38 – 0.72) | **1.24 (0.54 – 1.95)*** |
|  | c | 0.19 (-0.38 – 0.76) | **1.25 (0.52 – 1.98)*** |
| **NO_2_** | a | 0.21 (-0.13 – 0.55) | **0.89 (0.44 – 1.34)*** |
|  | b | 0.37 (-0.03 – 0.77) | **1.10 (0.57 – 1.62)*** |
|  | c | 0.42 (-0.01 – 0.84) | **1.11 (0.56 – 1.66)*** |
| **NO_x_** | a | 0.26 (-0.14 – 0.65) | **1.04 (0.52 – 1.56)*** |
|  | b | 0.44 (-0.02 – 0.89) | **1.27 (0.67 – 1.86)*** |
|  | c | **0.49 (0.01 – 0.97)** | **1.29 (0.66 – 1.91)*** |
| **O_3_** | a | **0.80 (0.30 – 1.31)*** | 0.13 (-0.64 – 0.89) |
|  | b | **0.77 (0.26 – 1.29)*** | 0.08 (-0.69 – 0.84) |
|  | c | **0.85 (0.31 – 1.39)*** | 0.12 (-0.67 – 0.89) |
| **Models** (a): unadjusted; (b): adjusted for NDVI300, deprivation index, ethnicity, sex and SES; (c): adjusted for Model b variables + distance to industry and type of residential area. Bold p= 0.05; *Multiple comparison p threshold= 0.012  The standardized increments for each exposure are as follows: 20 µg/m³ for NOx, 10 µg/m³ for NO_2_, PM_10_, and O_3_, 5 µg/m³ for PM_coarse_ and PM_2.5_, and 10⁻⁵ m⁻¹ for PM_2.5 abs_  **Abbreviations**: PM_2.5_: Particulate matter with aerodynamic diameter ≤ 2.5 μm; PM_10_: Particulate matter with aerodynamic diameter ≤ 10 μm; PM_coarse_: Particulate matter 2.5–10 μm; PM_2.5abs_: Absorbance of PM < 2.5 μm; NO_2_: Nitrogen dioxide; NO_x_: Nitrogen oxides; O_3_: Ozone. | | | |

| **Table 2:** Beta coefficient (β) with 95% confidence interval (CI) for the association between prenatal air pollutants and ADHD symptoms total score reported by *parents* for preschool-aged and school-age children | | | |
| --- | --- | --- | --- |
| **Pollutants** | **Models** | **Preschool-aged children** | **School-age children** |
|  |  | **β *(CI 95%)*** | **β *(CI 95%)*** |
| **PM_2.5_** | a | -0.39 (-2.11 – 1.32) | 0.14 (-2.01 – 2.28) |
|  | b | -0.18 (-1.93 – 1.56) | 0.71 (-1.44 – 2.86) |
|  | c | -0.26 (-2.03 – 1.51) | 0.68 (-1.56 – 2.92) |
| **PM_10_** | a | -0.15 (-1.37 – 1.03) | -0.44 (-1.84 – 0.97) |
|  | b | -0.75 (-2.01 – 0.51) | -0.35 (-1.85 – 1.14) |
|  | c | -0.78 (-2.09 – 0.53) | -0.34 (-1.86 – 1.19) |
| **PM_2.5abs_** | a | 0.00 (0.00 – 0.00) | 0.00 (0.00 – 0.00) |
|  | b | 0.00 (0.00 – 0.00) | 0.00 (0.00 – 0.00) |
|  | c | 0.00 (0.00 – 0.00) | 0.00 (0.00 – 0.00) |
| **PM_coarse_** | a | 0.16 (-0.58 – 0.90) | -0.04 (-0.92 – 0.84) |
|  | b | -0.17 (-0.93 – 0.59) | -0.08 (-0.97 – 0.80) |
|  | c | -0.22 (-1.00 – 0.56) | -0.11 (-1.03 – 0.80) |
| **NO_2_** | a | 0.05 (-0.41 – 0.52) | -0.04 (-0.62 – 0.53) |
|  | b | -0.11 (-0.65 – 0.43) | 0.26 (-0.39 – 0.93) |
|  | c | -0.13 (-0.72 – 0.44) | 0.31 (-0.38 – 1.03) |
| **NO_x_** | a | 0.06 (-0.47 – 0.59) | -0.03 (-0.69 – 0.63) |
|  | b | -0.12 (-0.74 – 0.50) | 0.31 (-0.45 – 1.06) |
|  | c | -0.15 (-0.81 – 0.50) | 0.36 (-0.43 – 1.15) |
| **O_3_** | a | 0.27 (-0.40 – 0.95) | 0.38 (-0.58 – 1.34) |
|  | b | 0.39 (-0.31 – 1.10) | 0.27 (-0.69 – 1.24) |
|  | c | 0.37 (-0.37 – 1.11) | 0.23 (-0.74 – 1.21) |
| **Models** (a): unadjusted; (b): adjusted for NDVI300, deprivation index, ethnicity, sex and SES; (c): adjusted for Model b variables + distance to industry and type of residential area. Bold p= 0.05; *Multiple comparison p threshold= 0.012  The standardized increments for each exposure are as follows: 20 µg/m³ for NOx, 10 µg/m³ for NO_2_, PM_10_, and O_3_, 5 µg/m³ for PM_coarse_ and PM_2.5_, and 10⁻⁵ m⁻¹ for PM_2.5 abs_  **Abbreviations**: PM_2.5_: Particulate matter with aerodynamic diameter ≤ 2.5 μm; PM_10_: Particulate matter with aerodynamic diameter ≤ 10 μm; PM_coarse_: Particulate matter 2.5–10 μm; PM_2.5abs_: Absorbance of PM < 2.5 μm; NO_2_: Nitrogen dioxide; NO_x_: Nitrogen oxides; O_3_: Ozone. | | | |

| **Table 3:** Beta coefficient (β) with 95% confidence interval (CI) for the association between prenatal air pollutants and ADHD symptoms total score reported by parents for preschool-aged and school-age children. | | | | | | | | | |
| --- | --- | --- | --- | --- | --- | --- | --- | --- | --- |
|  |  | **Preschool-aged children** | | | | **School-age children** | | | |
| **Pollutants** | **Models** | **Restless-impulsivity** | | **Emotional liability** | | **Hyperactivity-impulsivity** | | **Inattention** | |
|  |  | **β *(CI 95%)*** | | **β *(CI 95%)*** | | **β *(CI 95%)*** | | **β *(CI 95%)*** | |
|  |  | **Male**  **(n = 900)** | **Female**  **(n = 902)** | **Male**  **(n = 900)** | **Female**  **(n = 902)** | **Male**  **(n = 898)** | **Female**  **(n = 1027)** | **Male**  **(n = 898)** | **Female**  **(n = 1027)** |
| **PM_2.5_** | a | -0.02 (-0.19 – 0.16) | -0.11 (-0.31 – 0.09) | -0.01 (-0.19 – 0.17) | 0.03 (-0.16 – 0.23) | -0.03 (-0.22 – 0.15) | -0.03 (-0.18 – 0.12) | 0.03 (-0.15 – 0.21) | -0.01 (-0.16 – 0.14) |
|  | b | -0.03 (-0.22 – 0.15) | -0.06 (-0.26 – 0.15) | -0.04 (-0.23 – 0.14) | 0.11 (-0.09 – 0.30) | -0.01 (-0.19 – 0.18) | -0.04 (-0.19 – 0.12) | -0.06 (-0.12 – 0.25) | -0.02 (-0.14 – 0.17) |
|  | c | -0.04 (-0.23 – 0.15) | -0.06 (-0.27 – 0.14) | -0.06 (-0.25 – 0.13) | 0.11 (-0.09 – 0.32) | 0.01 (-0.18 – 0.21) | -0.01 (-0.17 – 0.15) | 0.09 (-0.09 – 0.29) | 0.01 (-0.16 – 0.17) |
| **PM_10_** | a | 0.01 (-0.11 – 0.13) | -0.02 (-0.16 – 0.11) | -0.09 (-0.22 – 0.03) | 0.03 (-0.10 – 0.16) | -0.06 (-0.18 – 0.06) | -0.03 (-0.13 – 0.07) | -0.04 (-0.15 – 0.08) | -0.04 (-0.14 – 0.06) |
|  | b | -0.02 (-0.16 – 0.11) | -0.03 (-0.17 – 0.12) | -0.16 (-0.29 – 0.02) | 0.01 (-0.13 – 0.15) | -0.05 (-0.17 – 0.08) | -0.04 (-0.15 – 0.06) | -0.01 (-0.13 – 0.12) | -0.02 (-0.13 – 0.08) |
|  | c | -0.02 (-0.16 – 0.12) | -0.03 (-0.18 – 0.12) | -0.17 (-0.32 – 0.02) | 0.12 (-0.13 – 0.16) | -0.05 (-0.18 – 0.07) | -0.04 (-0.15 – 0.08) | 0.01 (-0.11 – 0.14) | -0.04 (-0.15 – 0.08) |
| **PM_2.5abs_** | a | 0.00 (0.00 – 0.00) | 0.00 (0.00 – 0.00) | 0.00 (0.00 – 0.00) | 0.00 (0.00 – 0.00) | 0.00 (0.00 – 0.00) | 0.00 (0.00 – 0.00) | 0.00 (0.00 – 0.00) | 0.00 (0.00 – 0.00) |
|  | b | 0.00 (0.00 – 0.00) | 0.00 (0.00 – 0.00) | 0.00 (0.00 – 0.00) | 0.00 (0.00 – 0.00) | 0.00 (0.00 – 0.00) | 0.00 (0.00 – 0.00) | 0.00 (0.00 – 0.00) | 0.00 (0.00 – 0.00) |
|  | c | 0.00 (0.00 – 0.00) | 0.00 (0.00 – 0.00) | 0.00 (0.00 – 0.00) | 0.00 (0.00 – 0.00) | 0.00 (0.00 – 0.00) | 0.00 (0.00 – 0.00) | 0.00 (0.00 – 0.00) | 0.00 (0.00 – 0.00) |
| **PM_coarse_** | a | 0.03 (-0.06 – 0.10) | 0.02 (-0.06 – 0.10) | -0.04 (-0.12 – 0.34) | 0.04 (-0.05 – 0.12) | -0.01 (-0.07 – 0.07) | -0.05 (-0.12 – 0.01) | 0.01 (-0.06 – 0.08) | -0.01 (0.07 – 0.05) |
|  | b | 0.00 (-0.08 – 0.08) | 0.01 (-0.08 – 0.09) | -0.08 (-0.16 – 0.01) | 0.02 (-0.06 – 0.11) | -0.01 (-0.08 – 0.07) | -0.05 (-0.12 – 0.01) | 0.01 (-0.07 – 0.08) | -0.02 (-0.08 – 0.05) |
|  | c | -0.01 (-0.08 – 0.08) | 0.00 (-0.08 – 0.09) | -0.09 (-0.18 – 0.01) | 0.02 (-0.06 – 0.11) | -0.01 (-0.08 – 0.08) | -0.05 (-0.12 – 0.01) | 0.02 (-0.05 – 0.10) | -0.02 (-0.09 – 0.05) |
| **NO_2_** | a | 0.03 (-0.02 – 0.07) | -0.03 (-0.08 – 0.02) | -0.01 (-0.06 – 0.04) | 0.01 (-0.05 – 0.05) | -0.01 (-0.06 – 0.04) | -0.01 (-0.05 – 0.03) | 0.01 (-0.04 – 0.06) | -0.01 (-0.05 – 0.03) |
|  | b | 0.02 (-0.04 – 0.08) | -0.03 (-0.09 – 0.03) | -0.03 (-0.09 – 0.02) | -0.01 (-0.06 – 0.06) | 0.01 (-0.04 – 0.07) | -0.02 (-0.06 – 0.03) | 0.04 (-0.01 – 0.10) | 0.01 (-0.04 – 0.05) |
|  | c | 0.02 (-0.04 – 0.08) | -0.03 (-0.10 – 031) | -0.05 (-0.11 – 0.01) | 0.01 (-0.06 – 0.07) | 0.01 (-0.05 – 0.07) | -0.01 (-0.06 – 0.03) | 0.06 (-0.01 – 0.12) | -0.01 (-0.05 – 0.05) |
| **NO_x_** | a | 0.03 (-0.02 – 0.09) | -0.04 (-0.09 – 0.24) | -0.01 (-0.06 – 0.05) | 0.01 (-0.06 – 0.06) | -0.01 (-0.07 – 0.05) | -0.01 (-0.05 – 0.03) | 0.01 (-0.04 – 0.07) | -0.01 (-0.05 – 0.03) |
|  | b | 0.02 (-0.04 – 0.09) | -0.04 (-0.11 – 0.03) | -0.04 (-0.11 – 0.03) | -0.01 (-0.07 – 0.06) | 0.01 (-0.05 – 0.08) | -0.02 (-0.07 – 0.03) | 0.05 (-0.02 – 0.11) | 0.01 (-0.05 – 0.06) |
|  | c | 0.03 (-0.04 – 0.09) | -0.04 (-0.12 – 0.03) | -0.05 (-0.12 – 0.02) | 0.00 (-0.07 – 0.07) | 0.12 (-0.06 – 0.08) | -0.01 (-0.07 – 0.04) | 0.07 (-0.00 – 0.13) | -0.01 (-0.06 – 0.06) |
| **O_3_** | a | 0.02 (-0.04 – 0.09) | 0.00 (-0.08 – 0.08) | 0.07 (-0.01 – 0.14) | 0.02 (-0.06 – 0.09) | 0.10 (-0.02 – 0.18) | -0.02 (-0.04 – 0.09) | 0.01 (-0.07 – 0.09) | 0.04 (-0.03 – 0.11) |
|  | b | 0.04 (-0.04 – 0.11) | -0.02 (-0.11 – 0.06) | 0.09 (-0.02 – 0.16) | 0.01 (-0.21 – 1.69) | 0.07 (-0.01 – 0.16) | 0.03 (-0.04 – 0.09) | -0.01 (-0.09 – 0.07) | 0.04 (-0.03 – 0.11) |
|  | c | 0.04 (-0.04 – 0.11) | -0.02 (-0.11 – 0.06) | 0.08 (-0.01 – 0.17) | 0.01 (-0.08 – 0.08) | 0.08 (-0.01 – 0.17) | 0.03 (-0.04 – 0.10) | -0.01 (-0.09 – 0.07) | 0.04 (-0.03 – 0.11) |
| Linear regression models were used to estimate β coefficients and 95% CIs.  **Models** (a): unadjusted; (b): adjusted for NDVI300, deprivation index, ethnicity and SES; (c): adjusted for Model b variables + distance to industry and type of residential area. Bold p= 0.05; *Multiple comparison p threshold= 0.012  The standardized increments for each exposure are as follows: 20 µg/m³ for NOx, 10 µg/m³ for NO_2_, PM_10_, and O_3_, 5 µg/m³ for PM_coarse_ and PM_2.5_, and 10⁻⁵ m⁻¹ for PM_2.5 abs_  **Abbreviations**: PM_2.5_: Particulate matter with aerodynamic diameter ≤ 2.5 μm; PM_10_: Particulate matter with aerodynamic diameter ≤ 10 μm; PM_coarse_: Particulate matter 2.5–10 μm; PM_2.5abs_: Absorbance of PM < 2.5 μm; NO_2_: Nitrogen dioxide; NO_x_: Nitrogen oxides; O_3_: Ozone. | | | | | | | | | |

| **Table 4:** Odds Ratio (OR) with 95% confidence interval (CI) for the association between prenatal exposure to air pollutants and ADHD presentations. | | | | |
| --- | --- | --- | --- | --- |
| **Pollutants** | **Models** | ***Inattention*** | ***Hyperactivity*** | ***Combined*** |
|  |  | **OR *(CI 95%)*** | **OR *(CI 95%)*** | **OR *(CI 95%)*** |
| **PM_2.5_** | a | 1.62 (0.81 – 3.27) | 0.73 (0.27 – 1.95) | 1.35 (0.76 – 2.41) |
|  | b | 0.99 (0.46 – 2.14) | 1.07 (0.37 – 3.03) | 0.95 (0.49 – 1.81) |
|  | c | 1.01 (0.44 – 2.28) | 0.87 (0.29 – 2.55) | 0.86 (0.44 – 1.66) |
|  | d | 1.04 (0.38 – 2.96) | 0.64 (0.16 – 2.49) | 0.87 (0.37 – 2.05) |
| **PM_10_** | a | 1.44 (0.92 – 2.25) | 0.79 (0.40 – 1.54) | 1.32 (0.92 – 1.90) |
|  | b | 1.17 (0.69 – 1.96) | 1.01 (0.48 – 2.13) | 1.13 (0.75 – 1.71) |
|  | c | 1.13 (0.65 – 1.94) | 0.89 (0.42 – 1.92) | 1.05 (0.67 – 1.61) |
|  | d | 0.91 (0.47 – 1.78) | 0.53 (0.19 – 1.42) | 0.85 (0.50 – 1.45) |
| **PM_2.5abs_** | a | 1.00 (1.00 – 1.00) | 1.00 (1.00 – 1.00) | 1.00 (1.00 – 1.00) |
|  | b | 1.00 (1.00 – 1.00) | 1.00 (1.00 – 1.00) | 1.00 (1.00 – 1.00) |
|  | c | 1.00 (1.00 – 1.00) | 1.00 (1.00 – 1.00) | 1.00 (1.00 – 1.00) |
|  | d | 1.00 (1.00 – 1.00) | 1.00 (1.00 – 1.00) | 1.00 (1.00 – 1.00) |
| **PM_coarse_** | a | 1.33 (0.99 – 1.78) | 0.84 (0.54 – 1.29) | 1.14 (0.89 – 1.45) |
|  | b | 1.16 (0.85 – 1.60) | 0.86 (0.54 – 1.37) | 1.04 (0.81 – 1.34) |
|  | c | 1.16 (0.83 – 1.63) | 0.81 (0.50 – 1.30) | 1.05 (0.77 – 1.31) |
|  | d | 0.96 (0.64 – 1.45) | 0.60 (0.32 – 1.13) | 0.86 (0.62 – 1.19) |
| **NO_2_** | a | 1.07 (0.89 – 1.30) | 0.86 (0.64 – 1.16) | 1.13 (0.97 – 1.33) |
|  | b | 1.12 (0.89 – 1.41) | 1.02 (0.71 – 1.45) | 1.11 (0.91 – 1.34) |
|  | c | 1.09 (0.86 – 1.39) | 0.96 (0.66 – 1.40) | 1.07 (0.87 – 1.31) |
|  | d | 0.93 (0.69 – 1.25) | 0.75 (0.47 – 1.18) | 1.01 (0.79 – 1.28) |
| **NO_x_** | a | 1.08 (0.87 – 1.34) | 0.83 (0.59 – 1.17) | 1.15 (0.96 – 1.38) |
|  | b | 1.13 (0.86 – 1.47) | 1.01 (0.66 – 1.51) | 1.12 (0.89 – 1.39) |
|  | c | 1.10 (0.84 – 1.45) | 0.94 (0.62 – 1.44) | 1.07 (0.85 – 1.34) |
|  | d | 0.91 (0.65 – 1.28) | 0.70 (0.42 – 1.19) | 1.00 (0.75 – 1.32) |
| **O_3_** | a | 0.77 (0.57 – 1.04) | 0.79 (0.52 – 1.21) | 0.90 (0.72 – 1.14) |
|  | b | 0.99 (0.72 – 1.38) | 0.59 (0.36 – 0.97) | 1.05 (0.81 – 1.37) |
|  | c | 1.03 (0.73 – 1.43) | 0.59 (0.36 – 0.99) | 1.09 (0.83 – 1.44) |
|  | d | 1.11 (0.75 – 1.67) | 0.57 (0.32 – 1.04) | 1.20 (0.86 – 1.66) |
| **Models** (a): unadjusted; (b): adjusted for NDVI300, deprivation index, ethnicity and SES; (c): adjusted for Model b variables + distance to industry and type of residential area. Bold p= 0.05; *Multiple comparison p threshold= 0.012  The standardized increments for each exposure are as follows: 20 µg/m³ for NOx, 10 µg/m³ for NO_2_, PM_10_, and O_3_, 5 µg/m³ for PM_coarse_ and PM_2.5_, and 10⁻⁵ m⁻¹ for PM_2.5 abs_  **Abbreviations:** PM_2.5_: Particulate matter with aerodynamic diameter ≤ 2.5 μm; PM_10_: Particulate matter with aerodynamic diameter ≤ 10 μm; PM_coarse_: Particulate matter 2.5–10 μm; PM_2.5abs_: Absorbance of PM < 2.5 μm; NO_2_: Nitrogen dioxide; NO_x_: Nitrogen oxides; O_3_: Ozone. | | | | |

| **Table 5:** Beta coefficient (β) with 95% confidence interval (CI) for the association between prenatal air pollutants and ADHD hyperactivity-impulsivity score per trimester from Conners | | | | | |
| --- | --- | --- | --- | --- | --- |
| **Pollutants** | ***Models*** | **Trimester 1** | **Trimester 2** | **Trimester 3** | **Entire Pregnancy** |
|  |  | ***β (CI 95%)*** | ***β (CI 95%)*** | ***β (CI 95%)*** | ***β (CI 95%)*** |
| **PM_2.5_** | a | 0.07 (-0.01 – 0.16) | 0.05 (-0.03 – 0.13) | 0.03 (-0.05 – 0.11) | 0.12 (-0.01 – 0.24) |
|  | b | 0.08 (-0.01 – 0.16) | 0.03 (-0.04 – 0.11) | 0.02 (-0.05 – 0.10) | 0.10 (-0.02 – 0.22) |
|  | c | 0.08 (-0.01 – 0.16) | 0.03 (-0.05 – 0.11) | 0.02 (-0.06 - 0.09) | 0.10 (-0.02 – 0.23) |
| **PM_10_** | a | **0.09 (0.03 – 0.16)*** | **0.08 (0.02 – 0.14)*** | 0.06 (-0.01 – 0.11) | **0.13 (0.05 – 0.21)*** |
|  | b | **0.07 (0.01 – 0.14)** | 0.04 (-0.02 – 0.10) | 0.02 (-0.05 – 0.10) | **0.08 (0.01 – 0.17)** |
|  | c | **0.06 (0.01 – 0.13)** | 0.03 (-0.03 – 0.09) | 0.02 (-0.06 – 0.09) | 0.07 (-0.01 – 0.15) |
| **PM_2.5abs_** | a | 0.00 (0.00 – 0.00) | 0.00 (0.00 – 0.00) | 0.00 (0.00 – 0.00) | 0.00 (0.00 – 0.00) |
|  | b | 0.00 (0.00 – 0.00) | 0.00 (0.00 – 0.00) | 0.00 (0.00 – 0.00) | 0.00 (0.00 – 0.00) |
|  | c | 0.00 (0.00 – 0.00) | 0.00 (0.00 – 0.00) | 0.00 (0.00 – 0.00) | 0.00 (0.00 – 0.00) |
| **PM_coarse_** | a | **0.06 (0.02 – 0.11)*** | **0.05 (0.01 – 0.09)** | 0.04 (-0.01 – 0.08) | **0.07 (0.02 – 0.12)*** |
|  | b | **0.06 (0.01 – 0.09)** | 0.04 (-0.01 – 0.08) | 0.03 (-0.01 – 0.08) | **0.06 (0.01 – 0.11)** |
|  | c | **0.05 (0.01 – 0.09)** | 0.04 (-0.01 – 0.08) | 0.03 (-0.01 – 0.07) | **0.06 (0.01 – 0.11)** |
| **NO_2_** | a | **0.05 (0.02 – 0.08)*** | **0.05 (0.02 – 0.08) *** | **0.05 (0.02 – 0.08)** | **0.05 (0.02 – 0.08)*** |
|  | b | **0.04 (0.01 – 0.08)** | 0.04 (0.01 – 0.07) | **0.04 (0.01 – 0.07)** | **0.05 (0.01 – 0.08)*** |
|  | c | **0.03 (0.01 – 0.07)** | 0.03 (-0.01 – 0.07) | 0.03 (-0.01 – 0.07) | **0.04 (0.01 - 0.07)** |
| **NO_x_** | a | **0.06 (0.02 – 0.09)*** | **0.06 (0.02 – 0.09)** | **0.06 (0.02 – 0.09)** | **0.06 (0.03 – 0.09)*** |
|  | b | **0.05 (0.01 – 0.08)** | **0.04 (0.01 – 0.08)** | 0.04 (0.01 – 0.08) | **0.05 (0.01 – 0.09)** |
|  | c | **0.04 (0.00 – 0.08)** | 0.04 (-0.01 – 0.07) | 0.04 (-0.01 – 0.08) | 0.04 (-0.01 – 0.08) |
| **O_3_** | a | -0.02 (-0.04 – 0.01) | -0.01 (-0.03 – 0.02) | 0.01 (-0.02 – 0.03) | -0.03 (-0.08 – 0.02) |
|  | b | -0.02 (-0.04 – 0.01) | 0.01 (-0.02 – 0.02) | 0.01 (-0.01 – 0.03) | -0.03 (-0.08 – 0.03) |
|  | c | -0.02 (-0.04 – 0.01) | 0.01 (-0.02 – 0.02) | 0.01 (-0.01 – 0.03) | -0.02 (-0.07 – 0.04) |
| **Models** (a): unadjusted; (b): adjusted for NDVI300, deprivation index, age, sex, ethnicity and SES; (c): adjusted for Model b variables + distance to industry and type of residential area, toxins during pregnancy, low birth weight, weeks of pregnancy; (d): adjusted for Model c variables + ASRS risk. Bold p= 0.05; *Multiple comparison p threshold= 0.012  The standardized increments for each exposure are as follows: 20 µg/m³ for NOx, 10 µg/m³ for NO_2_, PM_10_, and O_3_, 5 µg/m³ for PM_coarse_ and PM_2.5_, and 10⁻⁵ m⁻¹ for PM_2.5 abs_  **Abbreviations:** PM_2.5_: Particulate matter with aerodynamic diameter ≤ 2.5 μm; PM_10_: Particulate matter with aerodynamic diameter ≤ 10 μm; PM_coarse_: Particulate matter 2.5–10 μm; PM_2.5abs_: Absorbance of PM < 2.5 μm; NO_2_: Nitrogen dioxide; NO_x_: Nitrogen oxides; O_3_: Ozone. | | | | | |

| **Table 6:** Beta coefficient (β) with 95% confidence interval (CI) for the association between prenatal air pollutants and ADHD hyperactivity-impulsivity severity score per trimester from K-SADS | | | | | |
| --- | --- | --- | --- | --- | --- |
| **Pollutants** | ***Models*** | **Trimester 1** | **Trimester 2** | **Trimester 3** | **Entire Pregnancy** |
|  |  | ***β (CI 95%)*** | ***β (CI 95%)*** | ***β (CI 95%)*** | ***β (CI 95%)*** |
| **PM_2.5_** | a | -0.31 (-1.03 – 0.42) | -0.17 (-0.87 – 0.52) | -0.12 (-0.80 – 0.56) | -0.41 (-1.41 – 0.58) |
|  | b | -0.24 (-0.99 – 0.51) | -0.03 (-0.76 – 0.68) | 0.05 (-0.65 – 0.75) | -0.13 (-1.22 – 0.95) |
|  | c | -0.33 (-1.09 – 0.44) | -0.07 (-0.79 – 0.66) | -0.02 (-0.73 – 0.68) | -0.27 (-1.39 – 0.85) |
|  | d | -0.49 (-1.51 – 0.53) | 0.02 (-0.94 – 0.98) | -0.09 (-0.99 – 0.82) | -0.47 (-1.97 – 1.04) |
| **PM_10_** | a | -0.05 (-0.59 – 0.48) | 0.03 (-0.49 – 0.56) | 0.02 (-0.51 – 0.56) | 0.01 (-0.65 – 0.67) |
|  | b | -0.06 (-0.64 – 0.52) | 0.09 (-0.47 – 0.66) | 0.10 (-0.45 – 0.66) | 0.09 (-0.64 – 0.83) |
|  | c | -0.14 (-0.73 – 0.46) | 0.04 (-0.53 – 0.62) | 0.04 (-0.53 – 0.61) | -0.02 (-0.78 – 0.75) |
|  | d | -0.52 (-1.29 – 0.26) | -0.08 (-0.84 – 0.67) | -0.23 (-0.96 – 0.51) | -0.49 (-1.50 – 0.51) |
| **PM_2.5abs_** | a | 0.00 (0.00 – 0.00) | 0.00 (0.00 – 0.00) | 0.00 (0.00 – 0.00) | 0.00 (0.00 – 0.00) |
|  | b | 0.00 (0.00 – 0.00) | 0.00 (0.00 – 0.00) | 0.00 (0.00 – 0.00) | 0.00 (0.00 – 0.00) |
|  | c | 0.00 (0.00 – 0.00) | 0.00 (0.00 – 0.00) | 0.00 (0.00 – 0.00) | 0.00 (0.00 – 0.00) |
|  | d | 0.00 (0.00 – 0.00) | 0.00 (0.00 – 0.00) | 0.00 (0.00 – 0.00) | 0.00 (0.00 – 0.00) |
| **PM_coarse_** | a | -0.05 (-0.43 – 0.33) | 0.02 (-0.36 – 0.39) | 0.03 (-0.35 – 0.40) | -0.01 (-0.44 – 0.42) |
|  | b | -0.09 (-0.49 – 0.29) | 0.01 (-0.38 – 0.39) | 0.02 (-0.37 – 0.41) | -0.03 (-0.48 – 0.42) |
|  | c | -0.15 (-0.55 – 0.25) | -0.04 (-0.43 – 0.36) | -0.03 (-0.43 – 0.36) | -0.09 (-0.55 – 0.37) |
|  | d | -0.41 (-0.94 – 0.12) | -0.14 (-0.66 – 0.39) | -0.23 (-0.75 – 0.28) | -0.37 (-0.99 – 0.25) |
| **NO_2_** | a | 0.12 (-0.15 – 0.38) | 0.16 (-0.11 – 0.43) | 0.14 (-0.14 – 0.42) | 0.15 (-0.13 – 0.44) |
|  | b | 0.17 (-0.14 – 0.48) | 0.23 (-0.07 – 0.55) | 0.22 (-0.09 – 0.54) | 0.25 (-0.08 – 0.59) |
|  | c | 0.11 (-0.22 – 0.43) | 0.18 (-0.14 – 0.50) | 0.16 (-0.17 – 0.49) | 0.19 (-0.16 – 0.55) |
|  | d | 0.03 (-0.39 – 0.45) | 0.20 (-0.22 – 0.63) | 0.13 (-0.29 – 0.55) | 0.13 (-0.32 – 0.58) |
| **NO_x_** | a | 0.14 (-0.17 – 0.44) | 0.18 (-0.12 – 0.49) | 0.16 (-0.16 – 0.48) | 0.18 (-0.15 – 0.50) |
|  | b | 0.19 (-0.16 – 0.55) | 0.27 (-0.09 – 0.63) | 0.26 (-0.09 – 0.63) | 0.28 (-0.09 – 0.67) |
|  | c | 0.12 (-0.25 – 0.49) | 0.20 (-0.17 – 0.57) | 0.18 (-0.19 – 0.56) | 0.22 (-0.18 – 0.62) |
|  | d | 0.03 (-0.46 – 0.52) | 0.22 (-0.27 – 0.72) | 0.15 (-0.33 – 0.64) | 0.14 (-0.38 – 0.66) |
| **O_3_** | a | -0.01 (-0.22 – 0.21) | 0.05 (-0.17 – 0.27) | -0.02 (-0.23 – 0.19) | 0.04 (-0.38 – 0.45) |
|  | b | -0.03 (-0.24 – 0.18) | 0.01 (-0.22 – 0.23) | -0.08 (-0.30 – 0.13) | -0.17 (-0.63 – 0.28) |
|  | c | -0.01 (-0.23 – 0.20) | 0.00 (-0.23 – 0.23) | -0.08 (-0.29 – 0.14) | -0.14 (-0.62 – 0.33) |
|  | d | -0.04 (-0.31 – 0.23) | -0.04 (-0.34 – 0.25) | -0.04 (-0.32 – 0.24) | -0.12 (-0.73 – 0.49) |
| **Models** (a): unadjusted; (b): adjusted for NDVI300, deprivation index, age, sex, ethnicity and SES; (c): adjusted for Model b variables + distance to industry and type of residential area, toxins during pregnancy, low birth weight, weeks of pregnancy; (d): adjusted for Model c variables + ASRS risk. Bold p= 0.05; *Multiple comparison p threshold= 0.012  The standardised increments for each exposure are as follows: 20 µg/m³ for NOx, 10 µg/m³ for NO_2_, PM_10_, and O_3_, 5 µg/m³ for PM_coarse_ and PM_2.5_, and 10⁻⁵ m⁻¹ for PM_2.5 abs_  **Abbreviations:** PM_2.5_: Particulate matter with aerodynamic diameter ≤ 2.5 μm; PM_10_: Particulate matter with aerodynamic diameter ≤ 10 μm; PM_coarse_: Particulate matter 2.5–10 μm; PM_2.5abs_: Absorbance of PM < 2.5 μm; NO_2_: Nitrogen dioxide; NO_x_: Nitrogen oxides; O_3_: Ozone. | | | | | |
